# Massive Loss of Proprioceptive Ia Synapses in Rat Spinal Motoneurons after Nerve Crush Injuries in the Postnatal Period

**DOI:** 10.1523/ENEURO.0436-22.2023

**Published:** 2023-02-14

**Authors:** Ariadna Arbat-Plana, Sara Bolívar, Xavier Navarro, Esther Udina, Francisco J. Alvarez

**Affiliations:** 1 Department of Cell Biology, Physiology and Immunology, Institute of Neurosciences, Universitat Autònoma de Barcelona, 08028 Barcelona, Spain; 2 Centro de Investigación Biomédica en Red sobre Enfermedades Neurodegenerativas (CIBERNED), 08193 Bellaterra, Spain; 3 Department of Physiology, Emory University, Atlanta, Georgia 30322

**Keywords:** Ia afferent, microglia, motoneuron, neonates, nerve injury, plasticity

## Abstract

Peripheral nerve injuries (PNIs) induce the retraction from the ventral horn of the synaptic collaterals of Ia afferents injured in the nerve, effectively removing Ia synapses from α-motoneurons. The loss of Ia input impairs functional recovery and could explain, in part, better recovery after PNIs with better Ia synaptic preservation. Synaptic losses correlate with injury severity, speed, and efficiency of muscle reinnervation and requires ventral microglia activation. It is unknown whether this plasticity is age dependent. In neonates, axotomized motoneurons and sensory neurons undergo apoptosis, but after postnatal day 10 most survive. The goal of this study was to analyze vesicular glutamate transporter 1 (VGluT1)-labeled Ia synapses (which also include II afferents) after nerve crush in 10 day old rats, a PNI causing little Ia/II synapse loss in adult rats. We confirmed fast and efficient reinnervation of leg muscles; however, a massive number of VGluT1/Ia/II synapses were permanently lost. This synapse loss was similar to that after more severe nerve injuries involving full transection in adults. In adults, disappearance of ventrally directed Ia/II collaterals targeting α-motoneurons was associated with a prolonged microglia reaction and a CCR2 mechanism that included infiltration of CCR2 blood immune cells. By contrast, microgliosis after P10 injuries was fast, resolved in about a week, and there was no evidence of peripheral immune cell infiltration. We conclude that VGluT1/Ia/II synapse loss in young animals differs in mechanism, perhaps associated with higher microglia synaptic pruning activity at this age and results in larger losses after milder nerve injuries.

## Visual Abstract

**Figure:**
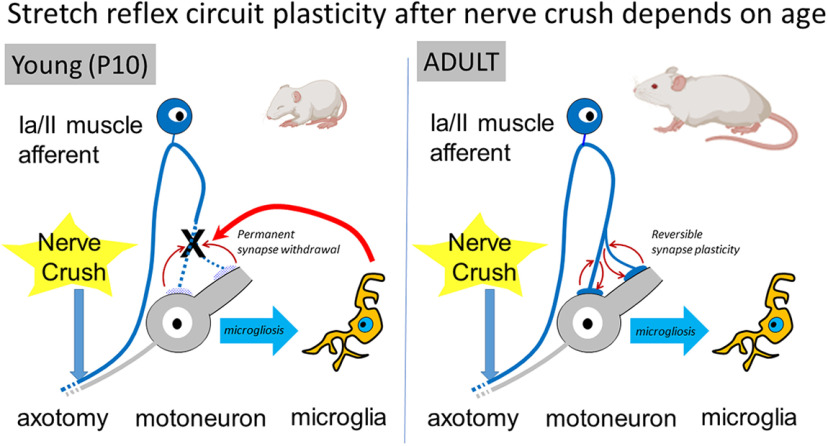

## Significance Statement

Synaptic plasticity in spinal motor circuits induced by nerve injuries can be rather permanent and hinder motor and sensory function recovery, even when experimental manipulations are designed for fast and efficient regeneration and target innervation. One input particularly affected is the Ia afferent synapse on α-motoneurons. The degree of loss of this input depends on injury severity and correlates with varying efficiencies while reinnervating peripheral targets. Here we demonstrate that the loss is also age dependent, being higher in the young. The enhanced synaptic pruning capabilities of microglia during critical windows of high synaptic plasticity at an early age might contribute to augment synaptic circuit reorganizations.

## Introduction

Outcomes after nerve injury depend on age and injury severity. In adults, injured sensory and motor axons regenerate and reinnervate their targets (Gordon, 2016), but while functional recovery after nerve crush is normal, recovery is poor after nerve transection (Navarro et al., 1994; Bodine-Fowler et al., 1997; Lundborg, 2000; Valero-Cabré and Navarro, 2001; Bontioti et al., 2003; Gordon, 2020). This is partly explained by connectivity changes after regeneration following nerve transections. Peripherally, axon misdirection degrades muscle specificity after nerve transection, but not after crush (Brushart and Mesulam, 1980; Molander and Aldskogius, 1992; Madison et al., 1996; Valero-Cabré et al., 2004; Gordon et al., 2009; Madison and Robinson, 2014; Bolívar et al., 2020). Additionally, nerve transections induce long-term changes in central synaptic circuits that contribute to dysfunction (Navarro et al., 2007; Alvarez et al., 2020).

Outcomes are different in young animals. Nerve injuries of any type induce loss of motoneurons (MNs) and sensory neurons in rodents of postnatal day 1 (P1) to P5 (Lowrie and Vrbová, 1992; Hart et al., 2008). Motoneuron loss is, however, absent when injuries occur after P7, although possible sensory neuron loss still occurs up to the second postnatal week (Kemp et al., 2015). Despite no or little cell loss, nerve crush at P11 or P12 impairs long-term motor function: muscle tension recovers to half of normal, with fast muscles more affected than slow muscles (Lowrie et al., 1987). Reduced functional recovery in the young despite efficient and precise muscle reinnervation (Nguyen et al., 2002) needs an explanation. The second postnatal week in the rat coincides with a critical window of synaptic plasticity underlying the maturation of weight-bearing motor function (Altman and Sudarshan, 1975; Westerga and Gramsbergen, 1990; Greensmith and Vrbová, 1992) and corresponds to the development of posture and limb coordination in 8- to 15-month-old human infants (Altman and Bayer, 2001). One possibility is that the developmental synaptic plasticity occurring during this period is disrupted by nerve crush inducing circuit alterations that do not occur in more mature synaptic circuits.

One input that undergoes significant changes after nerve injury in adults is that from Ia proprioceptors injured in the nerve (Alvarez et al., 2010, 2011; Bullinger et al., 2011; Rotterman et al., 2014). Ia afferents innervate muscle spindles and inform about muscle length dynamics (Matthews, 1981). Ia afferents synapse on α-motoneurons, mediating the stretch reflex, avoiding γ-motoneurons (Shneider et al., 2009). Irreversible loss of Ia afferent synaptic collaterals in the ventral horn after adult nerve transections abolishes the reflex and contributes to permanent motor dysfunction because diminished feedback information about muscles reaches α-motoneurons and spinal circuits coordinating motor function (Cope and Clark, 1993; Cope et al., 1994; Abelew et al., 2000; Haftel et al., 2005; Maas et al., 2007; Bullinger et al., 2011; Sabatier et al., 2011a,b; Horstman et al., 2019). In adults, Ia synapse losses are graded to injury severity with nerve transections causing large losses (Alvarez et al., 2011; Rotterman et al., 2014) and crush injuries no loss (Schultz et al., 2017), although Ia afferents are similarly axotomized. Correspondingly, Ia modulation of α-motoneurons and the stretch reflex are preserved after nerve crush in adults (Barker and Young, 1947; Prather et al., 2011). The fate of Ia synapses after nerve crush in younger animals is unknown.

Loss of Ia synapses after nerve transection depends on a spinal cord neuroinflammatory reaction that varies with injury severity (Rotterman et al., 2019). Nerve injuries causing maximal Ia afferent synapse plasticity associate with longer microglia activation, CCR2 activation, and infiltration of CCR2-expressing immune cells. Adult microglia are activated from a basal state devoted to synapse and neuron maintenance; however, postnatal microglia already actively express genes associated with synapse and axon pruning (Matcovitch-Natan et al., 2016; Hammond et al., 2019, 2021). Thus, nerve injury might influence microglia differently, depending on age. Indeed, dorsal horn microglia activation after “spared nerve injuries” leading to hyperalgesia, differs among neonatal, young, and adult rats (Vega-Avelaira et al., 2007). Similar age-related differences in ventral microglia could translate into more severe loss of Ia synapses after crush injuries in infants, a period of significant Ia afferent synapse pruning by microglia using complement mechanisms (Vukojicic et al., 2019). We therefore analyzed Ia–motoneuron synapses and microglia after nerve crush at P10 in rats. The results show larger synaptic losses than in adults and extensive microglia activation, but no infiltration of blood-derived immune cells. Ia synapse permanent loss after nerve crush in young rats might thus result from augmented microglia developmental synaptic pruning mechanisms.

## Materials and Methods

### Experimental animals

All experiments were conducted in postnatal Sprague Dawley rats of both sexes (*n* = 30) housed with their mothers until 21 d old, with free access to food and water at room temperature (22 ± 2°C) under a 12 h light/dark cycle. Tracer injections, when necessary, were performed before P10, and all nerve surgeries were performed at P10 (animal weight, 40–55 g). Recordings and tissue harvesting were performing at different times depending on the experimental groups described in Table 1. All procedures were approved by the local institutional ethics committee at the University of Barcelona and followed the guidelines of the European Commission on Animal Care (EU Directive 2010/63/EU) and the National Institutes of Health in the United States, and adhere to Society for Neuroscience policies.

**Table 1:** Experimental design: groups and follow-up time

Study	Follow-up
Group 1. Electrophysiology/analysis of end plates and microglia in the spinal cord in injured animals	7 dpi (–/*n* = 3)
	14 dpi (*n* = 18/3)
	21 dpi (*n* = 12/3)
	60 dpi (*n* = 5/2)
Group 2. VGluT1 along cell body and dendrite analyses (injured/naive)	7 dpi (*n* = 4/4)
	14 dpi (*n* = 4/4)
	60 dpi (*n* = 5/5)
Group 3. Percentage of proprioceptive neurons in L4 DRG in injured animals	14 dpi (*n* = 10)
Group 4. Analysis of microglia in the spinal cord in injured animals	3 dpi (*n* = 3)
	5 dpi (*n* = 3)

Animals were divided into four main experimental groups. In all of them except the naive group, the sciatic nerve was crushed. Group 1: electrophysiology study. A group of animals was followed up for 60 dpi to study CMAPs. To evaluate end plate reinnervation, 3 animals were killed for each time point. Group 2: analyses of VGluT1 distribution along dendrites animals were divided based on follow-up times of 7, 14, and 60 dpi. A subgroup of animals was used as a naive group (see text in results (*n* = 3)). Group 3: analysis of the percentage of proprioceptive neurons in L4 DRGs 2 weeks after injury. Contralateral DRGs were used as controls. Group 4: analysis of microglia reactivity in the spinal cord. Animals were divided based on follow-up times: 3, 5, 7, 14, and 21 dpi. For follow-ups at 7, 14, and 21 dpi, spinal cords from animals of group 1 were used.

### Retrograde labeling

To identify the gastrocnemius (G) motor pool on both sides of the spinal cord, the G muscles were bilaterally injected with retrograde tracers 2–3 d before P10. Depending on survival times, we used a short-term tracer [cholera subunit b coupled to Alexa Fluor 555; CTb-555 (CTb), Thermo Fisher Scientific] or a long-term tracer [Fast Blue (FB), Polysciences]. Two 1 μl injections of 1% CTb-555 diluted in sterile saline or 2.5% FB diluted in sterile water were injected at different locations into the body of the muscle with glass micropipettes (Sigma-Aldrich). CTb-555-injected animals were analyzed 1 and 2 weeks postinjury, and FB-injected animals were used for the 60 d survival time point.

### Nerve surgeries

P10 rats were anesthetized by administration of ketamine (0.3 ml/kg, i.p.; Imalgene 2000, Boehringer Ingelheim) and xylazine (0.17 ml/kg; Rompun 2%), and prepared for aseptic surgery. The sciatic nerve was exposed at mid-thigh and crushed during 30 s in three different orientations with a fine forceps. Afterward, the skin was closed, sutured, and iodine povidone applied to the wound. Pups recovered in a warm environment under close observation and were returned to the cage with the mother. Animals were studied at 3, 5, 7, 14, 21, and 60 d postsurgery, depending on experiments (Table 1).

### Electrophysiology tests

Motor reinnervation was assessed by measuring maximal compound muscle action potentials (CMAPs) at 14, 21, and 60 d postinjury (dpi). Electrophysiological evaluation was performed under anesthesia (ketamine, 0.9 ml/kg; xylazine, 0.5 ml/kg) and keeping body temperature constant between 34°C and 36°C by means of a thermostat-controlled flat coil. The sciatic nerve was stimulated percutaneously with two needle electrodes inserted at the sciatic notch. We applied single rectangular pulses of 0.1 ms duration and increasing intensity until the voltage required to obtain a maximal response. CMAPs were recorded from the tibialis anterior (TA) and G muscles with microneedle electrodes and an electromyography system (Synergy Medelec, Viasys HealthCare). All potentials were amplified, and the amplitude from baseline to maximal negative peak of the direct M wave was recorded for each animal. Measurements were done bilaterally to compare the injured side to the control side.

### Tissue harvesting for Histological processing and immunohistochemistry

At the end of survival periods the animals were deeply anesthetized with intraperitoneal pentobarbital (Dolethal; 30 mg/kg) and transcardially perfused with vascular rinse followed by 4% paraformaldehyde in 0.01 m PBS 0.9%. The L4 dorsal root ganglia (DRGs), L3 to L6 spinal cord segments, and selected muscles were removed and postfixed overnight. Tissue samples were cryoprotected and stored in 30% sucrose in PBS until used.

### End plate reinnervation of the G and TA muscles

G and TA muscles were quickly frozen in Tissue-Tek OCT (optimal cutting temperature compound; Sakura), and cryostat sections (20 μm thick) were collected on Fisherbrand Superfrost Plus glass slides (Thermo Fisher Scientific). Slides were kept at −20°C until used. Labeling was performed on the slides after bringing them to room temperature and washing the OCT with multiple changes in PBS. Thereafter, the sections were blocked with 10% normal donkey serum diluted in PBS with 0.3% Triton X-100 (PBST) for 1 h and incubated overnight at room temperature in Alexa Fluor 647-conjugated α-bungarotoxin (α-btx; 1:100; Thermo Fisher Scientific) and a mixture of primary antibodies that included guinea pig anti-vesicular acetylcholine transporter (VAChT; 1:200; EMD Millipore; RRID:AB_11214110) and phosphorylated chicken anti-neurofilament heavy chain (NFH; 1:200; Aves Labs; RRID:AB_2313552). The following day, the slides were washed in PBS and incubated for 2 h at room temperature with donkey anti-guinea pig Ig coupled to Cy3 and donkey anti-chicken IgY coupled to fluorescein isothiocyanate (FITC; 1:200; Jackson ImmunoResearch). After final washes in PBS, the sections were coverslipped with Vectashield (Vector Laboratories).

Sections were imaged in a confocal microscope (model FV1000, Olympus) at high magnification (60×; *z*-step size, 1 μm), capturing overlapping image tiles to sample a large proportion of the muscles neuromuscular junction (NMJ) fields. Reinnervation of α-btx-labeled neuromuscular junctions was analyzed in three animals per time point. For each muscle, at least 50 end plates per animal were imaged at 7, 14, and 21 dpi. At 60 dpi, we captured 15 α-btx-labeled neuromuscular junctions per animal. We considered a neuromuscular junction reinnervated when VAChT immunofluorescence was present. Full reinnervation was defined as more than 50% α-btx-labeled neuromuscular junction covered by motor end plate VAChT immunoreactivity, and partial reinnervation if VAChT immunoreactivity only covered some spots of the α-btx field. The percentage of α-btx neuromuscular junctions partially or fully reinnervated were estimated for each muscle and time point.

### Analyses of vesicular glutamate transporter 1 contacts on retrogradely labeled α-motoneurons

Vesicular glutamate transporter 1 (VGluT1) contacts on retrogradely labeled G motoneurons were analyzed at 7, 15, and 60 dpi in, respectively, four, four, and five animals (Table 1). The contralateral uninjured side was used as a control. To validate the uninjured side as control, it was first compared with motoneurons in three naive animals. In all cases, transverse spinal cords were obtained at 50 μm thickness in a sliding freezing microtome and processed for VGluT1 immunofluorescence. The sections were collected free floating, and, after blocking with 10% normal donkey serum for 1 h, they were incubated overnight in a solution containing rabbit anti-VGluT1 (1:1000; Synaptic Systems; RRID:AB_887877) diluted in PBST. The following day, sections were washed in PBS and incubated for 2 h in donkey anti-rabbit IgG antibodies conjugated to FITC (1:100; Jackson ImmunoResearch) in PBST. Slides were then washed in PBS, mounted serially on glass slides, and coverslipped with Vectashield (Vector Laboratories). Retrogradely labeled motoneurons (CTb-555 or FB) were localized, and images were captured with a confocal microscope (model FV1000, Olympus) using a 10× objective for neuron localization and a 60× objective (numerical aperture 1.35, oil-immersion) to image VGluT1 contacts along the somatodendritic surface.

Confocal images *z*-stacks were uploaded to Neurolucida (version 10.0; MBF Bioscience) to trace the cell bodies and dendrites to their ends within the 50 μm section (most dendrites are cut in these preparations, but they permit enough sampling of the proximal dendritic arbor where most VGluT1 contacts are located). The cell body was reconstructed through a series of contours traced in each optical plane. Dendrites were traced in 3D. Synaptic locations were marked in the traced neurons and VGluT1 synaptic densities analyzed using NeuroExplorer (version 10.0, MBF Bioscience). Morphological features including cell body surface, volume, cell body diameters, number of primary dendrites, and total length of individual and all dendritic segments contained within the section were measured. Only the most relevant measurements are reported. Motoneurons with cross-sectional cell body average diameter of <25 μm were not included in the analyses because they could belong to γ-motoneurons, which lack VGluT1 synapses (Shneider et al., 2009). To obtain VGluT1 densities, the number of VGluT1 contacts was divided by estimates of cell body surface, total dendrite surface, total dendrite length, or the surface of individual dendrite segments at different locations from the cell body. The densities of synapses at different distances from the cell body were estimated using path distance measurements or Sholl analyses. The results were similar. We considered that path–distance analyses were more rigorous than Sholl analyses because they take into account dendrite tortuosity; thus, the confirmatory Sholl analyses are not included. The traced dendritic arbor was divided into segments at path distances 0–50 , 50–100, or 100–150 μm from the cell body. We did not analyze segments beyond 150 μm because they had little representation in our sample. This is explained by the limitations of retrograde labeling and dendrites cut at the plane of section. We analyzed 4–6 α-motoneurons (average ± SD, 5.00 ± 0.57) per animal and side (contralateral or ipsilateral to the injury) for a total of 130 reconstructed α-motoneurons from 13 animals.

### Quantification of proprioceptive sensory neurons in dorsal root ganglia

To confirm cellular preservation of proprioceptive sensory neurons, we counted parvalbumin (PV)-immunoreactive (IR) cellular profiles in dorsal root ganglia (DRGs) ipsilateral and contralateral to the nerve crush. We analyzed L4 DRGs from 10 animals with a crush injury of the right sciatic nerve 14 d before. The DRGs were embedded in Tissue-Tek OCT, serially cut (15 μm thickness) with a cryostat, and collected onto gelatin-coated glass slides. All sections were first blocked with 10% normal bovine serum for 1 h, followed by overnight incubation at 4°C with primary antibodies, as follows: rabbit anti-parvalbumin (1:1000; catalog #PV28, Swant; RRID:AB_2315235); and mouse biotinylated anti-NeuN (1:200; catalog #MAB377B, Millipore; RRID:AB_177621). After washes, donkey anti-rabbit IgG Alexa Fluor 488 (1:200; catalog #A-21206, Thermo Fisher Scientific; RRID:AB_2535792) and Alexa Fluor 488-streptavidin (1:200; catalog #S32356, Thermo Fisher Scientific) were used to reveal IR sites. After 2 h of incubation at room temperature, the sections were thoroughly washed, mounted on slides, and coverslipped with Fluoromount-G (SouthernBiotech). DRG sections were visualized with a fluorescence microscope (model BX51, Olympus); at least four sections per DRG were captured at 40× with a digital camera (model DP50, Olympus) and CellSens Digital Imaging software (version 1.9; Olympus), and were merged using ImageJ software. The number of positive parvalbumin and all NeuN neurons were manually counted. NeuN was used to ensure that all cellular profiles counted were from mid-cell cross sections that included the nucleus. We then estimated the proportion of NeuN^+^ neurons that were parvalbumin^+^ in DRGs ipsilateral and contralateral to the nerve crush. This procedure normalized differences in cell numbers depending on the level and orientation of L4 DRG section cut. We analyzed four to five sections per DRG in six control L4 DRGs with an average (±SD) of 1560.0 ± 398.9 neurons sampled per ganglia and eight injured L4 DRGs with an average of 1533.6 ± 282.4 neurons per ganglia. Ganglia in which sectioning and immunocytochemistry processing did not allow recovery of four to five sections with >200 NeuN neurons per section were not included in the quantitative analysis to avoid errors in percentage estimates because of uneven clustering of parvalbumin-IR neurons in DRG or other possible biases in sections with small numbers of neurons.

### Quantification of neuroimmune cells

Loss of Ia/VGluT1 synapses requires a microglia reaction (Iba1^+^, CD11b^+^ cells) and activation of CCR2, which is also associated with infiltration of CD45^+^ CCR2^+^ CD11b^+^ peripheral macrophages and CD45^+^ CCR2^+^ CD3e^+^ T cells (see Introduction). The microglia reaction was analyzed by counting all Iba1^+^ cells in the ventral horn of the spinal cord at 3 d (*n* = 3), 5 d (*n* = 3), 7 d (*n* = 4), 14 d (*n* = 4), 21 d (*n* = 4), and 60 d (*n* = 2) after nerve crush injury at P10. To test for the presence of peripheral immune cells we tested for Iba1^+^, CD11b^+^, and/or CD45^+^ cells at 7, 14, 21, and 60 d after the injury. Transverse sections (50 μm thick) of L4-5 spinal cord were obtained in a sliding freezing microtome, collected free floating, blocked for 1 h with 10% normal donkey serum diluted in PBST, and incubated overnight at room temperature in a mixture containing a goat polyclonal antibody against Iba1 (1:500; Novus Biologicals; RRID:AB_521594) and a rat monoclonal antibody against CD11b (1:50; OX-43; catalog #M1/70.15.11.5.2-s, Developmental Hybridoma Bank) or a rabbit polyclonal antibody against CD45 (1:50; Abcam; RRID:AB_442810). Sections were washed in PBS and incubated for 2 h in donkey anti-goat IgG antibody conjugated to FITC and donkey anti-rat or anti-rabbit IgG conjugated to Cy3 (all secondary antibodies diluted 1:200 in PBST; Jackson ImmunoResearch). The sections were mounted on slides, coverslipped with Vectashield (Vector Laboratories), and imaged throughout with a confocal microscope (model FV1000, Olympus) using 1 μm *z*-steps and a 20× objective. Overlapping image tiles were captured to sample the whole spinal cord. At least three sections were imaged for each animal. We found no CD45^+^ cells, and therefore quantitative analyses focused on Iba1^+^ microglia. The ventral horn of each hemisection was manually selected by marking a region of interest (ROIs) limited dorsally by a straight horizontal line above the central canal and in all other directions by the border between gray and white matter. All Iba1^+^ cells inside these ROIs were counted using ImageJ software. Because the spinal cord changes in size with maturation, we calculated cell densities, as follows: the number of Iba1^+^ microglia divided by the ventral horn volume calculated from the ROI area multiplied by section thickness (50 μm). Densities ratios were obtained for each section between the side ipsilateral to the injury and the contralateral control side.

### Statistical analysis

Normality was assessed with the Shapiro–Wilk test, and usually the data were compared using one-way or two-way ANOVA to test for significant differences in time after injury and lesion versus contralateral control side when appropriate, and any possible interactions. *Post hoc* pairwise comparisons were performed using Bonferroni-corrected *t* tests. If normality was not satisfied, we used an ANOVA on ranks (with some exceptions indicated in the text) and *post hoc* Dunn’s tests. DRG numbers in control versus injured sides at a single time point were compared using a *t* test. Statistical analyses were performed in SigmaPlot (version 12) or Prism (version 9). Correlations between functional (CMAPs) and anatomical reinnervation were fitted in SPSS 20.0. A value of *p *<* *0.05 was considered significant. The power of performed tests was always >0.8 for α = 0.05, unless indicated in text. When power was not reached because effect sizes were too small, we performed estimation statistics and a permutation *t* test to report effect sizes when the null hypothesis of zero difference is rejected. For each permutation *p* value, we performed 5000 bootstrap reshuffles of the control and injured sides, and the data are represented with a Gardner–Altman estimation plot (Ho et al., 2019). Small skews in the distribution of mean differences were overcome using a bias-corrected and accelerated bootstrap method.

## Results

### Functional muscle motor reinnervation after nerve crush injury at P10

**Figure 1.**
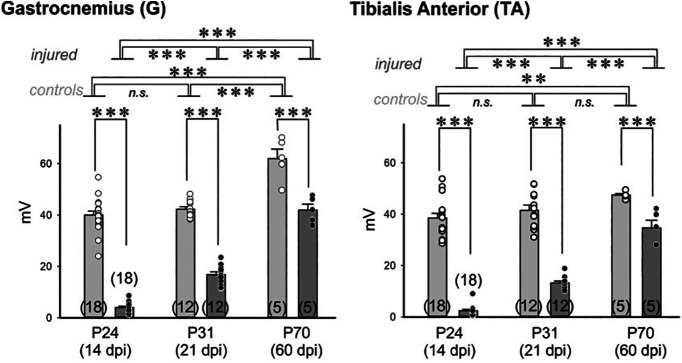
Electromyographic testing of muscle responses before and after nerve injury. Results of CMAPs recorded in G and TA muscles at 14, 21, and 60 dpi in the control and injured side. Controls are in light gray and injured animals in medium gray. Data are expressed in absolute values as the mean ± SEM. Each dot represents the average CMAP value in a different animal. The number of animals tested is at the bottom of the bar. A two-way ANOVA for injured versus control and time after injury showed significant differences according to both variables and their interactions (all *p* < 0.001). *Post hoc* Bonferroni corrected *t* tests showed significant differences with development and injury as indicated in the figure: **p < 0.01, ***p < 0.001. n.s., Nonsignificant. Full statistics are shown in Extended Data Figure 1-1.

Electromyographic recordings of the G and TA muscles were performed in the injured and control sides of rats with a unilateral sciatic nerve crush at P10. To evaluate the progression of motor reinnervation, we examined animals at 14 dpi (*n* = 18 animals), 21 dpi (*n* = 12), and 60 dpi (*n* = 5; Fig. 1, Extended Data Fig. 1-1). The progressive decrease in sample size with increased time is explained by a proportion of animals being killed at each time point for tissue harvesting to examine NMJ reinnervation and/or microglia in the spinal cord. No analyses were performed at 7 dpi, since at this time no evoked responses were recorded and anatomically there was almost no NMJ reinnervation (see below). The maximal CMAP (or muscle, M response) recorded in the G muscle of the intact hindlimb after stimulation of the ipsilateral uninjured sciatic nerve had mean ± SEM amplitudes of 40.0 ± 6.7 and 42.3 ± 3.1 mV at, respectively, P24 and P31 in age (corresponding to 14 and 21 dpi of the contralateral injured sciatic nerve), whereas at P70 (60 dpi) it was higher (62.0 ± 8.1 mV). In the intact TA muscles, the increase in CMAP amplitude was more gradual (from 38.6 ± 6.7 mV at P24 to 47.4 ± 1.4 mV at P70). CMAPs in the injured side were always lower compared with the contralateral control side at any time in both muscles. Two-way ANOVAs for injury and age showed significant differences according to both variables and their interaction (*p* < 0.001) in both G and TA (although normality failed in the Shapiro–Wilk test, we chose to do parametric tests since zeros at the early reinnervation dates lead to the loss of normality in the distribution). *Post hoc* pairwise Bonferroni’s *t* tests (Extended Data Fig. 1-1) showed that at P70, CMAPs in the intact hindlimb G were significantly higher than at P24 or P31 (*p* < 0.001). In contrast, CMAP amplitude in the intact TA was significantly higher comparing P70 to P24 (*p* = 0.004), but not comparing P31 with either. This suggests that G showed a faster developmental increase of CMAP amplitudes during the first month compared with TA.

10.1523/ENEURO.0436-22.2023.f1-1 Figure 1-1 Statistical table for CMAP comparisons at different postinjury dates. Download Figure 1-1, DOCX file.

CMAPs were always significantly lower in the injured side compared with age-matched controls in both G and TA (*p* < 0.001). In G, CMAP mean amplitude at 14 dpi (P24) was 10% of control, increasing to 40% of control at 21 dpi (P31) and 68% at 60 dpi (P70; Extended Data Fig. 1-1). Similar patterns were observed in TA: limited recovery detected at 14 dpi, averaging 6.5% of control amplitude, increased at 21 dpi to 32% and to 73% at 60 dpi. Thus, G and TA recovered functional innervation in parallel, despite a faster developmental increase in CMAP amplitudes in G compared with TA.

### Motor axon reinnervation of neuromuscular junctions

**Figure 2.**
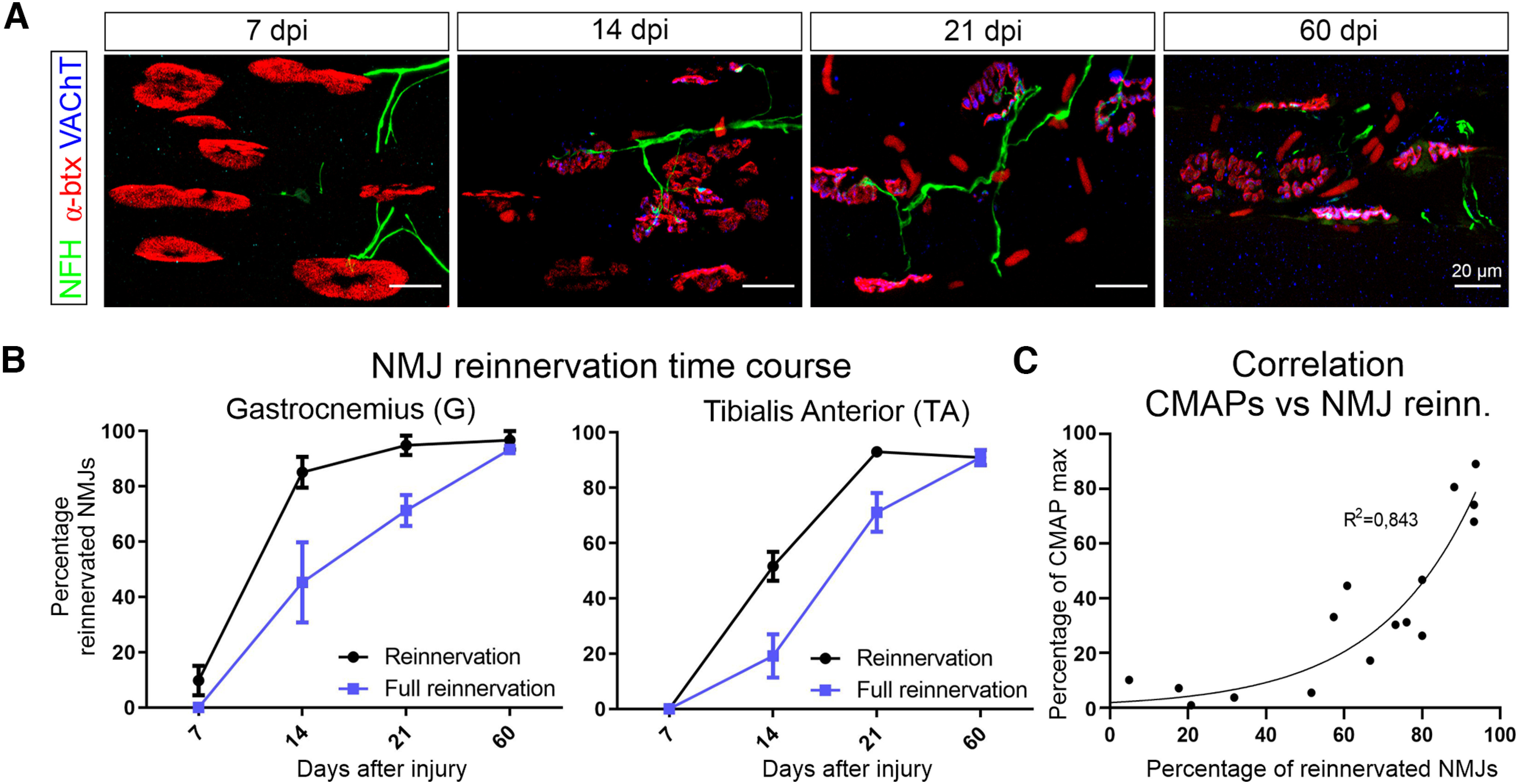
Anatomical reinnervation of the end plates. ***A***, Representative images of sections of a G muscle 7, 14, 21, and 60 dpi, immunostained against NFH, α-btx, and VAChT, respectively, to label axons, neuromuscular junctions, and the synaptic vesicle-filled motor end plate. Larger numbers of reinnervated NMJs were observed with increased time after injury, with almost complete innervation at 60 dpi. At 7 dpi, there is little innervation by motor end plates of vacated NMJs, although some axons reach some α-btx-labeled NMJs. The nicotinic receptor field expands after early denervation. ***B***, Percentages of reinnervated NMJs with time after injury in TA and G muscles. Average ± SEM of estimates of NMJ coverage in each animal (for both muscles: *n* = 3 at 7, 14, and 21 dpi; *n* = 2 at 60 dpi). Reinnervation indicates NMJs in which NFH axons with or without some VAChT superimposed onto the NMJ are present. Full reinnervation indicates when most of the NMJ area is covered by VAChT immunoreactivity. Statistics were performed on fully reinnervated percentages averaged from 3 animals at each age and 2 animals at 60 dpi. Error bars indicate the SEM. A one-way ANOVA for time after injury showed a significant change in reinnervation in both TA and G with time after injury (TA, *p* < 0.0001; G, *p* = 0.0008; Extended Data Fig. 2-1). *Post hoc* pairwise comparisons showed significant changes among all ages in TA, except for 7 versus 14 dpi (slow initial reinnervation) and 21 versus 60 dpi (reinnervation plateau). The number of reinnervated NMJs in G was significantly different at all ages compared with 7 dpi (faster reinnervation), and then it was significant in alternate dates (14 vs 60 dpi) but not consecutive dates (suggesting progressive reinnervation). All comparisons are shown in Extended Data Figure 2-1. Asterisks were omitted from the figure for clarity. ***C***, Correlation between the percentage of the muscle (M) response (CMAPs from Fig. 1) and the percentage of NMJs “fully” reinnervated fitted by a simple exponential regression with *R*^2^ = 0.843 and was significant at *p* < 0.001. M response recovery was delayed with respect to the initiation of anatomic reinnervation of motor end plates.

Functional reinnervation was compared with anatomical reinnervation of G and TA NMJs labeled with Alexa Fluor 555-conjugated α-btx. Motor axons were labeled with antibodies against phosphorylated NFH and motor end plates with VAChT antibodies (Fig. 2*A*). Muscles were analyzed at 7, 14, 21, and 60 dpi, respectively, in three, three, three, and two animals. The time course of partial or full reinnervation was faster in G compared with TA (Fig. 2*B*). At 7 dpi, some NFH-stained axons were seen close to denervated NMJs, but none of the TA NMJs were reinnervated by VAChT (total, 230 NMJs) and only 9.8 ± 5.4% (±SEM) of NMJs in the G (total, 144 NMJs) showed some signs of reinnervation where VAChT immunofluorescence partially covered the NMJ. Denervated NMJs at this time had ovoid shapes, quite different from the typical “pretzel-like” appearance of intact NMJs or NMJs denervated in mature adult rats. This suggests a faster structural disorganization of the postsynaptic acetylcholine receptor field after denervation at this early age. The presence of some VAChT in motor end plates opposite to α-btx-labeled NMJs increased significantly in both muscles at 14 dpi (G: 85.1 ± 5.6%, *n* = 167; TA: 51.6 ± 5.3%, *n* = 202), reaching a plateau at 21 dpi (G: 94.9 ± 3.5%, *n* = 157; TA: 93.1 ± 2.0%, *n* = 162) and 60 dpi (G: 96.7 ± 3.3%, *n* = 35; TA: 91.0 ± 2.8%, *n* = 33). “Full” reinnervation of NMJs was evaluated when VAChT immunofluorescence covered more than half of the NMJ-labeled receptor field. Fully innervated end plates increased in both muscles at 14 dpi (G, 45.3 ± 14.5%; TA, 19.1 ± 7.8%) and 21 dpi (G, 71.3 ± 5.3%; TA, 71.3 ± 7.0%), and was quite complete at 60 dpi (G, 93.3 ± 3.3%; TA, 91.0 ± 2.8%).

10.1523/ENEURO.0436-22.2023.f2-1 Figure 2-1 Statistical table for NMJ reinnervation at different postinjury dates. Download Figure 2-1, DOCX file.

Full NMJ reinnervation at each postinjury date was statistically compared using one-way ANOVA with average reinnervation estimates for each animal. Percentage values in animal averages closely parallel those calculated when pooling all NMJs from different animals together. As expected, the increase in the proportion of fully reinnervated NMJs in TA and G with time after injury was significant (TA, *p* < 0.001; G, *p* < 0.001; Extended Data Fig. 2-1). *Post hoc* Bonferroni’s *t* tests demonstrated significant differences in alternate dates and sometimes in consecutive dates, suggesting progressive increases in innervation (Extended Data Fig. 2-1). Importantly, NMJ reinnervation and CMAP amplitudes were correlated in those animals in which both parameters were simultaneously measured (*p* < 0.001, *R*^2^ = 0.843); however, the relationship was nonlinear (Fig. 2*C*). Functional recovery was delayed with respect to anatomical reinnervation and thereafter grew exponentially. At the study endpoint (P70 at 60 dpi), CMAP amplitudes slightly lagged behind toward full recovery despite >90% anatomical reinnervation.

### VGluT1 contacts on α-motoneuron cell bodies and dendrites

**Figure 3.**
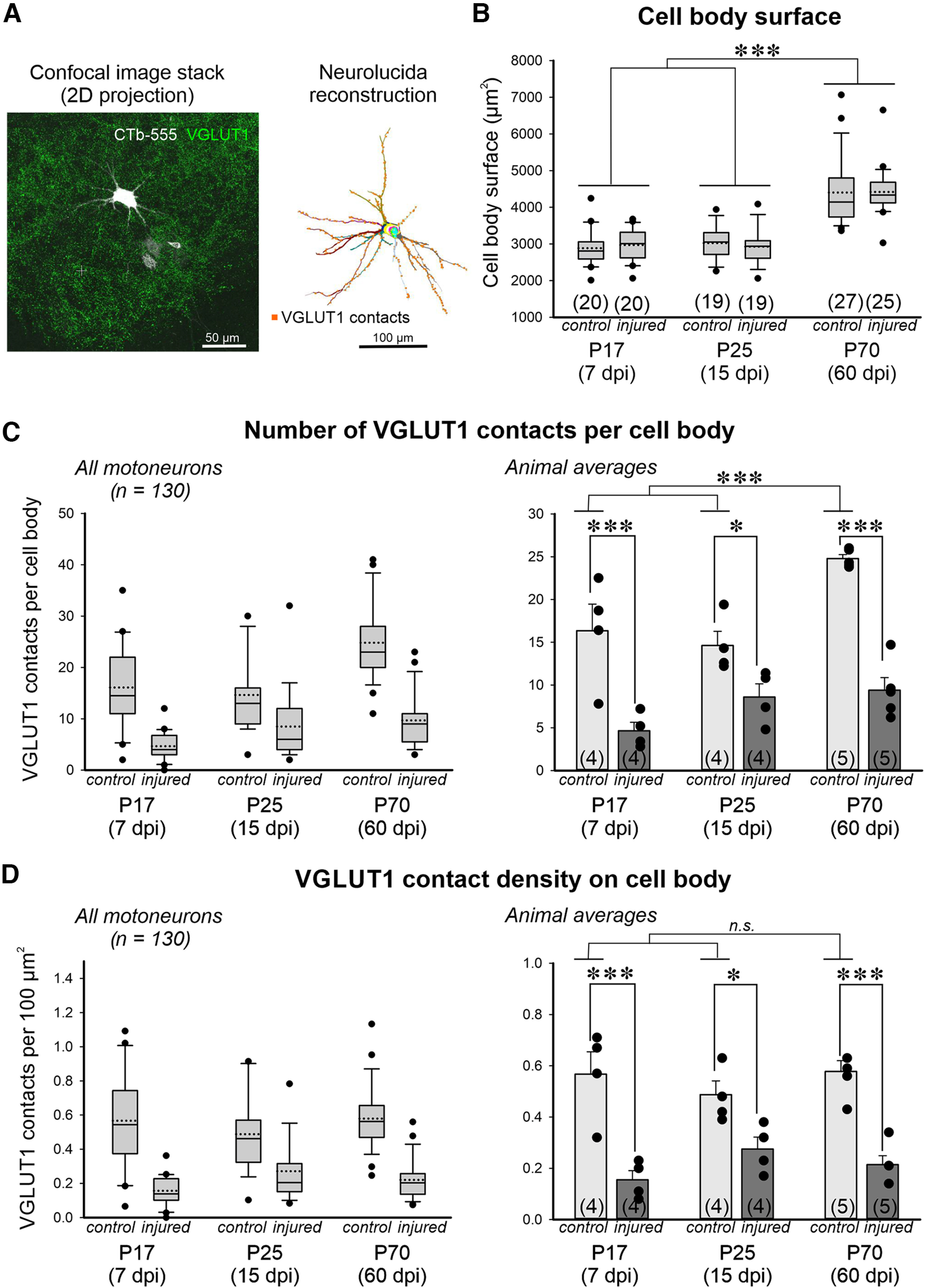
VGluT1 synapses on the cell bodies of injured α-motoneurons are significantly reduced compared with controls and are not recovered after muscle reinnervation. ***A***, Representative 2D projection image of a confocal stack (left) and Neurolucida reconstruction (right) of an Alexa Fluor 555 CTb-labeled motoneuron (white in confocal image) and VGluT1 boutons (green in confocal image). In the Neurolucida reconstruction, each dendrite and each optical cell body plane are in a different color. Yellow triangles indicate VGluT1 contacts on the cell body. Orange squares are VGluT1 contacts on dendrites. ***B***, Cell body surfaces of all reconstructed α-motoneurons at different ages and different times postinjury. Boxes represent data between the 25th and 75th percentiles with a continuous line within the box marking the median and a dashed line marking the average. Whiskers above and below the box indicate the 90th and 10th percentiles. Dots represent motoneuron surface estimates beyond these distribution boundaries. In brackets is the total sample of motoneurons reconstructed pooled from 4 animals at 7 and 14 dpi and from 5 animals at 60 dpi (4–6 motoneurons reconstructed per animal). Two-way ANOVA for differences according to injury and date showed significant differences only according to date and no interaction; pairwise comparisons comparing the three dates within controls or injured motoneurons showed statistically significant larger cell bodies at P70. ***p < 0.001 from *post hoc* Bonferroni’s *t* tests (Extended Data Fig. 3-1). ***C***, VGluT1 contacts per α-motoneuron cell body, by pooling all motoneurons (left) or calculated by animal averages (right). ***D***, VGluT1 density on cell bodies pooling all motoneurons (left) or by animal averages (right). Box plots in ***C*** and ***D*** represent data the same as in ***B*** and show the sample distributions. Per animal data are represented as the mean ± SEM, with each dot representing the average of each animal (number of animals in each condition/date are indicated in brackets). Two-way ANOVA revealed significant differences in the number of VGluT1 contacts according to time points (age/dpi, *p* = 0.0012), injury (*p* < 0.0001), or interaction between time point and injury (*p* = 0.0318). *Post hoc* Bonferroni’s corrected *t* tests for pairwise comparisons are indicated in the figure: *p < 0.05, **p < 0.01, ***p < 0.001. They show a significant increase in VGluT1 contact number at P70 in controls, but not injured motoneurons and significant decreases comparing injury and control at each time point (Extended Data Fig. 3-2). However, two-way ANOVA of VGluT1 densities resulted in a significant difference according to injury (*p* < 0.0001), but not time point (*p* = 0.792), and no interaction between the two (*p* = 171). Bonferroni’s corrected *t* tests of control versus injury at each day postinjury showed significant differences, as represented in the graph, but no difference in density across different time points for either control or injured data (Extended Data Fig. 3-2).

The above studies confirmed efficient muscle reinnervation 2 months after a sciatic nerve crush injury at P10. To establish the status of Ia afferent synapses on regenerating α-motoneurons, we analyzed VGluT1 synaptic contacts on 3D Neurolucida reconstructions of the cell body and dendrites of lateral G (LG) α-motoneurons retrogradely labeled with cholera toxin subunit b coupled to Alexa Fluor 555 (CTb-MNs) or with FB-MNs at 7, 14, and 60 dpi (Fig. 3*A*). Synaptic densities were compared with similarly retrogradely labeled α-motoneurons on the contralateral side and used as controls. To validate contralateral α-motoneurons as reliable controls, VGluT1 contacts on the cell body of LG CTb-MNs were compared between naive rats at P27 to P17 and P25 LG CTb-MNs contralateral to nerve injuries (respectively, 7 and 15 dpi). VGluT1 density on the cell body of CTb-MNs in P27 naive animals (*n* = 3 animals) was 0.47 +/– 0.38 VGluT1 contacts per 100 μm^2^ (±SD; *n* = 18 CTb-MNs), and on the control side of injured animals at P17 (*n* = 20 CTb-MNs) and P25 (*n* = 19 CTb-MNs) was respectively 0.57 ± 0.28 and 0.49 ± 0.22. Differences were not significant (*p* = 0.336; ANOVA on-ranks, H = 2.184 with 2 df).

10.1523/ENEURO.0436-22.2023.f3-1 Figure 3-1 Statistical table for changes in cell body total surface with age. Download Figure 3-1, DOCX file.

10.1523/ENEURO.0436-22.2023.f3-2 Figure 3-2 Statistical table for changes in cell body VGluT1 contact number and density according to age and/or injury. Download Figure 3-2, DOCX file.

The time of the analyses corresponds with a period of neuronal growth in the spinal cord. To estimate developmental changes in neuronal structure that occur independent of the injury, we analyzed cell body sizes. Cell body total surface estimates of CTb-MNs and FB-MNs (*n* = 130 motoneurons) did not change from P17 (7 dpi) to P25 (15 dpi), but increased in size (48% change) by P70 (60 dpi; Fig. 3*B*; mean ± SEM at P70 = 4410.1 ± 107.9 μm^2^; *n* = 52 FB-MNs). We did not detect significant differences in cell body size between control and age-matched injured CTb-MNs or FB-MNs at any age. Two-way ANOVA for age and injury demonstrated significant differences according to age (*p* < 0.001), but not according to injury (*p* = 0.947) or the interaction between age and injury (*p* = 0.761; Extended Data Fig. 3-1). *Post hoc* Bonferroni’s *t* tests showed significant differences at P70 with P17 and P25 (*p* < 0.001), but not between P17 and P25 (Fig. 3*B*, Extended Data Fig. 3-1). While control CTb-MNs showed a range of 2.234 and 1.679 μm^2^ in surface at P17 and P25, this increased to 3.708 μm^2^ at P70 in FB-MNs; however, the coefficient of variation remained relatively constant (P17, 5.9; P25, 5.5; P70, 4.9). In conclusion, developmental cell body growth includes proportional spreading of size distributions with age (and thus available surface for synaptic contacts), regardless of injury status.

The number of VGluT1 contacts was significantly decreased on the cell bodies of injured α-motoneurons compared with controls at all postinjury ages when pooling α-motoneurons from all animals (Fig. 3*C*, left). At 7, 15, and 60 dpi, we found a decrease in VGluT1 contacts on the cell body of, respectively, 71%, 42%, and 61%. Similar results were obtained when comparing average densities obtained per animal (Fig. 3*C*, right, Extended Data Fig. 3-2). A two-way ANOVA on animal averages indicated significant differences according to age (*p* = 0.0012), injury (*p* < 0.001), and their interaction (*p* = 0.032). *Post hoc* Bonferroni’s *t* tests on animal averages showed that at every age the decrease in VGluT1 contacts was significant (at 7 and 60 dpi, *p* < 0.0001; at 25 dpi, *p* = 0.022), In addition, the number of VGluT1 contacts increased with age in controls, and this was significant for P70 (60 dpi) compared with younger ages (*p* < 0.001), but was not significant between P17 and P24 (Fig. 3*C*, Extended Data Fig. 3-2). Interestingly, the age increase in VGluT1 contacts matched the increase in available cell body surface at P70. Thus, two-way ANOVA of VGluT1 densities (number of VGluT1 contacts per 100 μm^2^) showed significant differences with injury (*p* < 0.001), but not with age (*p* = 0.792), and differences found with injury were not significantly affected by age (*p* = 0.171). The percentage decreases in VGluT1 density (7 dpi, 72%; 14 dpi, 45%; 60 dpi, 62%) were similar to the percentage loss calculated for VGluT1 contacts per cell body and *post hoc* Bonferroni *t* tests on the averages of the animal showed that differences in VGluT1 densities were all consistently significant (at 7 and 60 dpi, *p* < 0.001; 15 dpi, *p* = 0.032). The results suggest that VGluT1 synapses are permanently removed from the cell bodies of α-motoneurons axotomized after crush at P10 and that the normal developmental addition of VGluT1 synapses to match the growth in available surface does not occur after crush.

**Figure 4.**
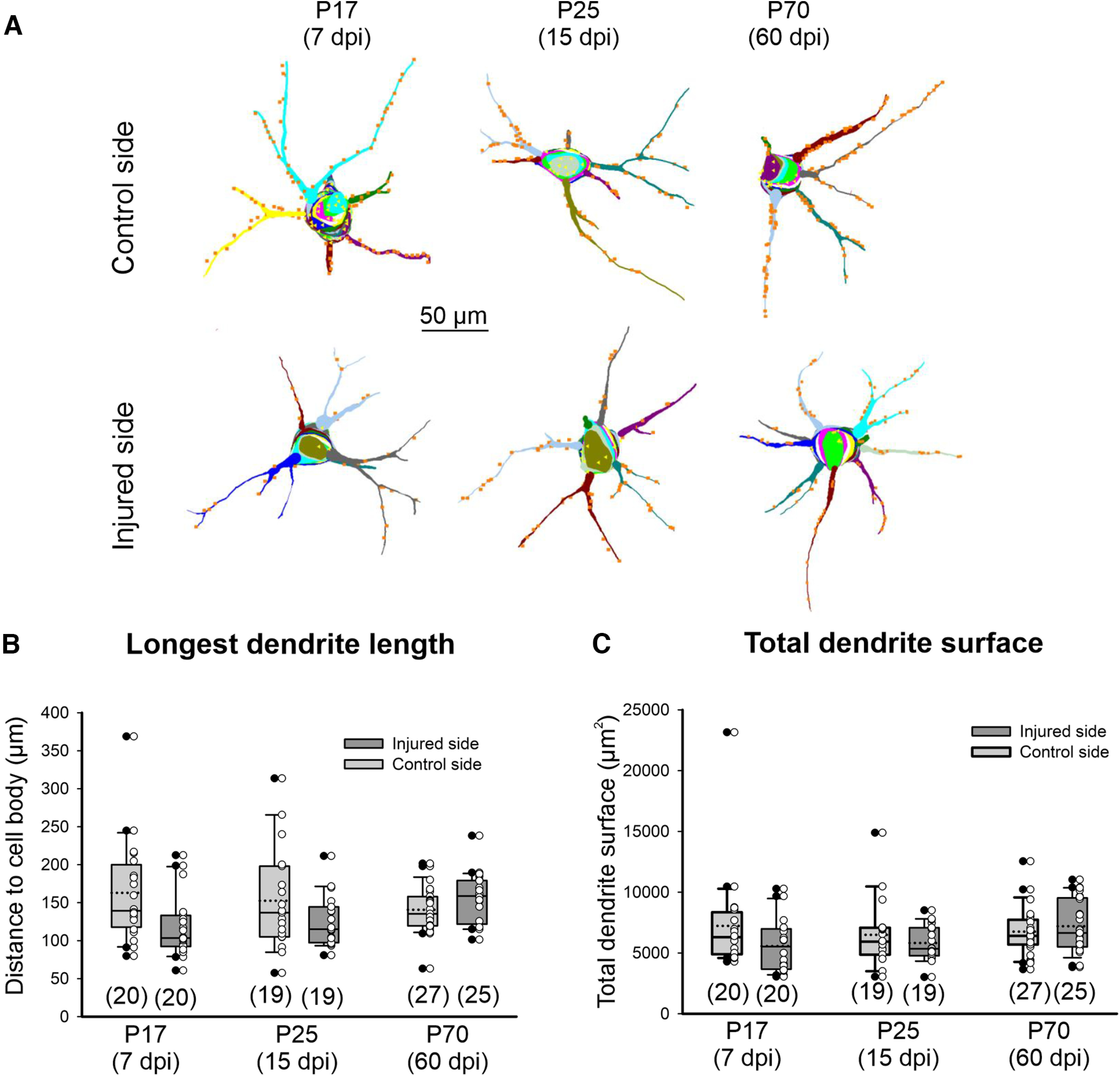
Sample properties of dendrite reconstructions. ***A***, Representative 3D Neurolucida reconstructions of control and injured α-motoneurons at each time point. Each dendrite and each optical cell body plane are in a different color. Yellow triangles indicate VGluT1 contacts on the cell body. Orange squares are VGluT1 contacts on dendrites. ***B***, Box plots representing the longest path distance of dendrite from the cell body analyzed for each α-motoneuron. Box: 25th and 75th percentiles with a continuous line within the box marking the median and a dashed line marking the average. Whiskers above and below the box indicate the 90th and 10th percentiles. Dots represent data points beyond these distribution boundaries. Light gray boxes are control α-motoneurons, darker gray boxes are injured α-motoneurons. Superimposed white dots represent individual α-motoneuron data. The large majority of motoneurons did not have dendritic segments farther than 200 μm, and within individual motoneurons many dendrites were traced for <150 μm path distance from the cell body. Two-way ANOVA showed no significant differences according to age/tracer (*p* = 0.756, *F*_(2_,_129)_ = 0.281) or injury (*p* = 0.117, *F*_(2_,_129)_ = 2.183). ***C***, Box plots representing the dendritic surface analyzed for each motoneuron. Despite a few outliers, our α-motoneuron sample did not significantly differ in the amount of dendritic surface analyzed in the different experimental groups (*p* = 0.169, *F*_(5,129)_ = 1.584).

However, the majority of VGluT1 synapses target α-motoneuron dendrites, in particular, the proximal dendritic arbor (Rotterman et al., 2014). VGluT1 depletions on dendrites usually differ in magnitude compared with changes on cell bodies (Alvarez et al., 2011; Rotterman et al., 2014; Schultz et al., 2017; Rotterman et al., 2019). Thus, we analyzed VGluT1 contacts along the retrogradely labeled dendritic arbors (Fig. 4*A*). Retrograde labeling preferentially labels the proximal dendritic tree, and no differences were found in the longest dendrite path distance to cell body or surface area measured in injured α-motoneurons compared with controls or at different ages (Fig. 4*B*,*C*). The known growth of the dendritic arbor from P15 to P70 (Kalb, 1994) affects more distal regions, and no differences were detected in our samples because the limited spread of the retrograde tracer to more proximal dendritic regions. Since all P70 α-motoneurons were traced with FB and all P17 and P25 α-motoneurons with CTb, the tracer variable was embedded with age. Two-way ANOVA showed no significant differences according to age/tracer (*p* = 0.756, *F*_(2_,_129)_ = 0.281) or injury (*p* = 0.117, *F*_(2_,_129)_ = 2.183). Similarly, there were no statistically significant differences on the total dendritic surface analyzed in the different experimental groups (*p* = 0.169, *F*_(5,129)_ = 1.584). Thus, both retrograde tracers labeled similar proximal regions of dendrite independent of age or injury, and by difference in analyses on the cell body, there were no age-related differences on average surface analyzed in different dendritic samples.

We decided to focus the study on the VGluT1 input to the first 150 μm of path distance from the cell body. This cutoff distance was chosen because it was reasonably represented in all our α-motoneuron samples; 85% of the sample included dendritic points farther than 100 μm from the cell body, but only 39% farther than 150 μm (*n* = 130 motoneurons in the total sample). For each motoneuron, we calculated VGluT1 contact numbers and surface densities in the following three distance segments: 0–50, 50–100, and 100–150 μm, with 0 being defined as the dendritic origin from the cell body (point of larger convexity between cell body and dendrite membranes). The distribution of VGluT1 synapses and their changes after injury in these distance bins were analyzed by measuring path distances (the distance to the cell body along the center of the traced dendrite) or by Sholl analyses (estimating the length or surface of the dendrite contained with concentric spheres at different distances from the cell body). Similar results were obtained with either method. This should be expected because α-motoneuron dendrites fan out in a radial manner and therefore there should be little difference between the methods. We report here only on path distance results that we consider more rigorous than Sholl analyses, since it also considers any tortuosity in dendrite trajectories.

**Figure 5.**
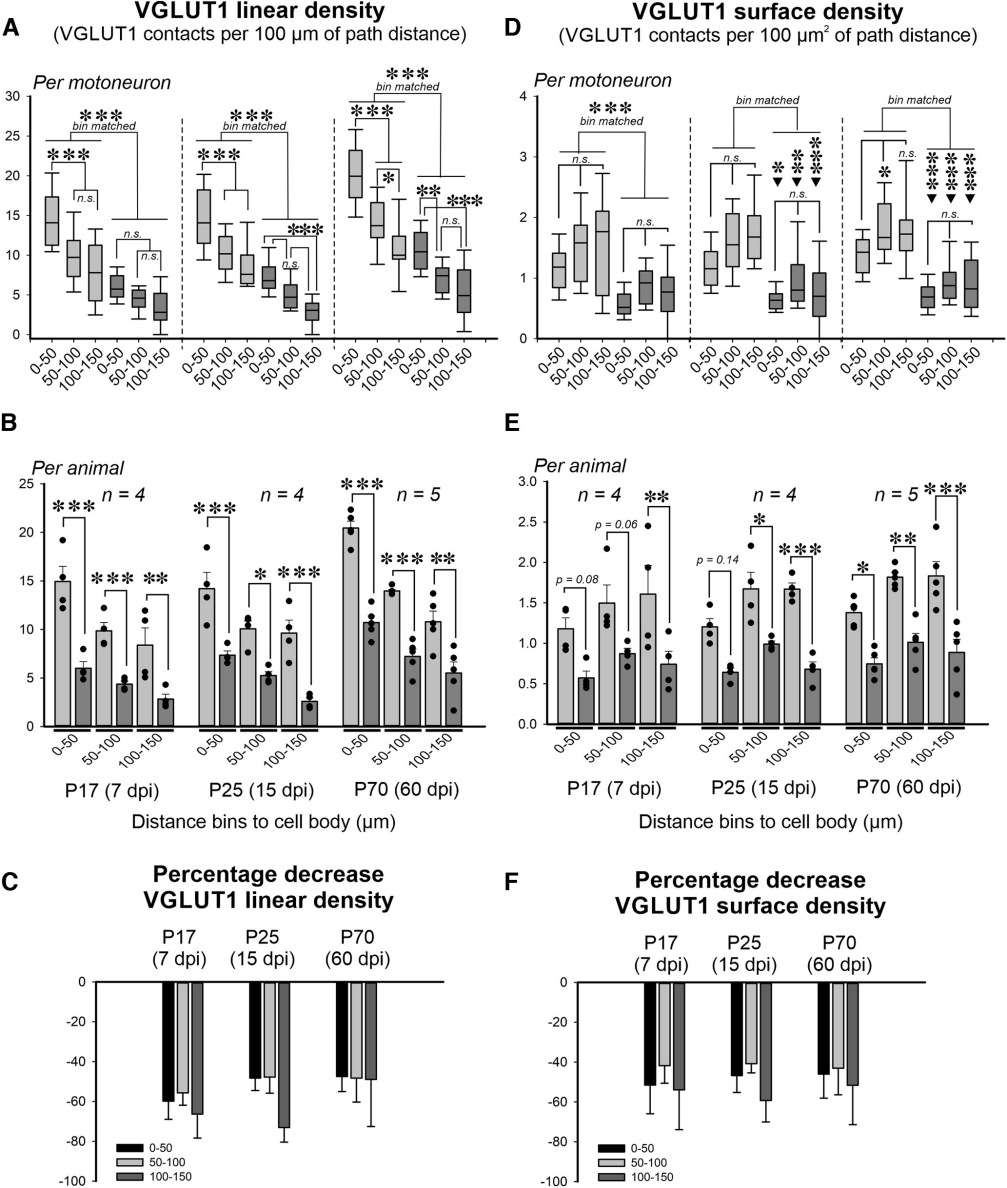
VGluT1 synapses on the dendrites of injured and control α-motoneurons. ***A–C***, Changes in VGluT1 linear density (number of VGluT1 contacts per 100 μm of dendritic length) according to injury, time point, and dendrite path distance to the cell body, either per α-motoneuron analyzed (***A***), animal averages (***B***), or percentage change comparing injured motoneurons to the average control (***C***). Box plots in ***A*** are as in previous figures: box represent the 25th to 75th percentile, line represents the median, whiskers represent the 10th to 90th percentiles. Bars in ***B*** represent the average VGluT1 linear density ± SEM; *n* = number animals. Dots represent each individual animal. In ***C***, the percentage change of VGluT1 linear density shows control versus injured motoneurons. In both ***A*** and ***B***, two-way ANOVAs showed statistically significant differences by injury/age and distance, but no interaction between the two (Extended Data Figs. 5-1, 5-2). In all graphs, statistical significance from pairwise *post hoc* Bonferroni’s *t* tests is expressed as follows: *p < 0.05, **p < 0.01, ***p < 0.001. *Post hoc* Bonferroni-corrected tests indicate significant depletions of VGluT1 contacts at each dendritic bin at all time points after injury. There is also a progressive and significant reduction in VGluT1 synapse numbers with distance to the cell body in controls. Finally, there is a significant increase in the number of contacts in most dendritic bins of control and injured α-motoneurons at P70 [two-way ANOVAs for dendrite compartment and age in either control or injured motoneurons; significance in changes with age at each dendritic bin is not depicted in graphs for clarity, but is fully described in Results and in Extended Data Figures 5-5 (control) and 5–6 (injured)]. ***D***, ***E***, Same representations as in ***A*** and ***B***, but for VGluT1 densities (number of VGluT1 contacts per 100 μm^2^ of dendrite surface). Similar two-way ANOVAs followed by *post hoc* Bonferroni’s tests demonstrate that VGluT1 densities are significantly reduced in each dendritic bin and in all ages, but VGluT1 density does not show significant differences with distance to the cell body (Extended Data Figs. 5-3, 5-4). The same trend is observed when considering individual motoneurons data (***D***) or animal averages (***E***), but significance in proximal dendritic compartments between control and injured α-motoneurons at P17 (7 dpi) was lost when pooling data in animal averages. ***F***, Similar to ***C*** (on VGluT1 numbers), the percentage decrease in VGluT1 density on all dendrite regions is always ∼50%.

VGluT1 input was calculated as linear density (number of contacts per 100 μm of dendrite length; Fig. 5*A–C*) or as surface density (number of contacts per 100 μm^2^ of dendrite surface; Fig. 5*D–F*). In control MNs, VGluT1 linear densities progressively decreased with distance to the cell body (Fig. 5*A*,*B*); however, VGluT1 surface densities were unchanged (Fig. 5*D*,*E*). This suggests that the decrease in VGluT1 contacts with distance matches the reduction in surface resulting from dendritic tapering further from the cell body. Two-way ANOVA on VGluT1 linear or surface densities for time point in control and injured α-motoneurons versus dendritic distance bins (0–50, 50–100, or 100–150 μm) showed significant differences with time point/injury and dendrite compartment (time point/injury, *p* < 0.0001; dendrite compartment: linear density, *p* < 0.001; surface density, *p* = 0.0067), but no interaction between the two (linear, *p* = 0.085; surface, 0.986). *Post hoc* pairwise comparisons showed significant differences between control and injured α-motoneurons in VGluT1 linear or surface density at each dendritic distance (Fig. 5*A*,*D*, Extended Data Figs. 5-1, 5-3), and this conclusion was, typically, replicated when animal averages were used (Fig. 5*B*,*E*, Extended Data Figs. 5-2, 5-4). The exception was for surface density differences between control and injury calculated from animal averages in the 0–50 μm distance bin at P17 and P25 and in the 100-150 distance bin at P17 (Fig. 5*E*). These bins showed clear reductions but did not reach significance in Bonferroni-corrected *t* tests of animal averages, although they did when all α-motoneurons for each experimental condition (age/injury status) were pooled together (Fig. 5*D*). These bins also showed significant differences when analyzed as linear densities whether using animal averages or pooling all α-motoneurons together. The discrepancy is best interpreted as a loss of statistical power with smaller differences detected when estimating surface densities versus number of contacts combined with the smaller sample size when considering *n* as the number of animals.

10.1523/ENEURO.0436-22.2023.f5-1 Figure 5-1 Statistical table for changes in dendrite VGluT1 linear density according to age, injury, and distance from the cell body. Download Figure 5-1, DOCX file.

10.1523/ENEURO.0436-22.2023.f5-2 Figure 5-2 Statistical table for changes in dendrites VGluT1 linear density according to age, injury, and distance from the cell body. Download Figure 5-2, DOCX file.

10.1523/ENEURO.0436-22.2023.f5-3 Figure 5-3 Statistical table for changes in dendrite VGluT1 surface density according to age, injury, and distance from the cell body. Download Figure 5-3, DOCX file.

10.1523/ENEURO.0436-22.2023.f5-4 Figure 5-4 Statistical table for changes in dendrite VGluT1 surface density according to age, injury, and distance from the cell body. Download Figure 5-4, DOCX file.

We also found a significant addition of synapses with age in the 0–50 and 50–100 μm distance compartments in controls (Fig. 5*A*, Extended Data Fig. 5-5; *post hoc* Bonferroni’s tests: *p* < 0.001 for P17 vs P70 and P25 vs P70 in both compartments) and injured α-motoneurons (Fig. 5*A*, Extended Data Fig. 5-6; *post hoc* Bonferroni’s tests: *p* < 0.001, P17 vs P70 in both dendritic compartments, and P25 vs P70 in the more proximal compartment; *p* = 0.0174, P25 vs P70 in the second compartments). This suggests that VGluT1 synapses are added in both dendrite regions throughout this postnatal period, but the developmental synaptic additions are smaller for injured α-motoneurons. In controls, the estimated difference between P25 and P70 in the number of VGluT1 contacts per 100 μm of dendrite was 4.71 ± 1.21 contacts ± SEM at 0–50 μm distance and 4.05 ± 1.10 contacts at 50–100 μm distance. In contrast, in injured α-motoneurons the estimated differences were 3.40 ± 0.70 at 0–50 μm and 2.00 ± 0.70 at 50–100 μm. In conclusion, α-motoneuron dendrites showed after nerve crush at P10 a significant loss of synapses and fewer synaptic additions with age. As a result, linear and surface densities were similarly reduced by approximately ≥50% in all dendrite distance bins of injured α-motoneurons compared with controls (Fig. 5*C*,*F*). Since by P70 there is ample motoneuron regeneration, these data suggest that injury-induced reduction in VGluT1 synapses is unrecoverable after muscle reinnervation.

10.1523/ENEURO.0436-22.2023.f5-5 Figure 5-5 Statistical table for changes in the number of VGluT1 contacts with age in each dendrite compartment in controls. Download Figure 5-5, DOCX file.

**Figure 6.**
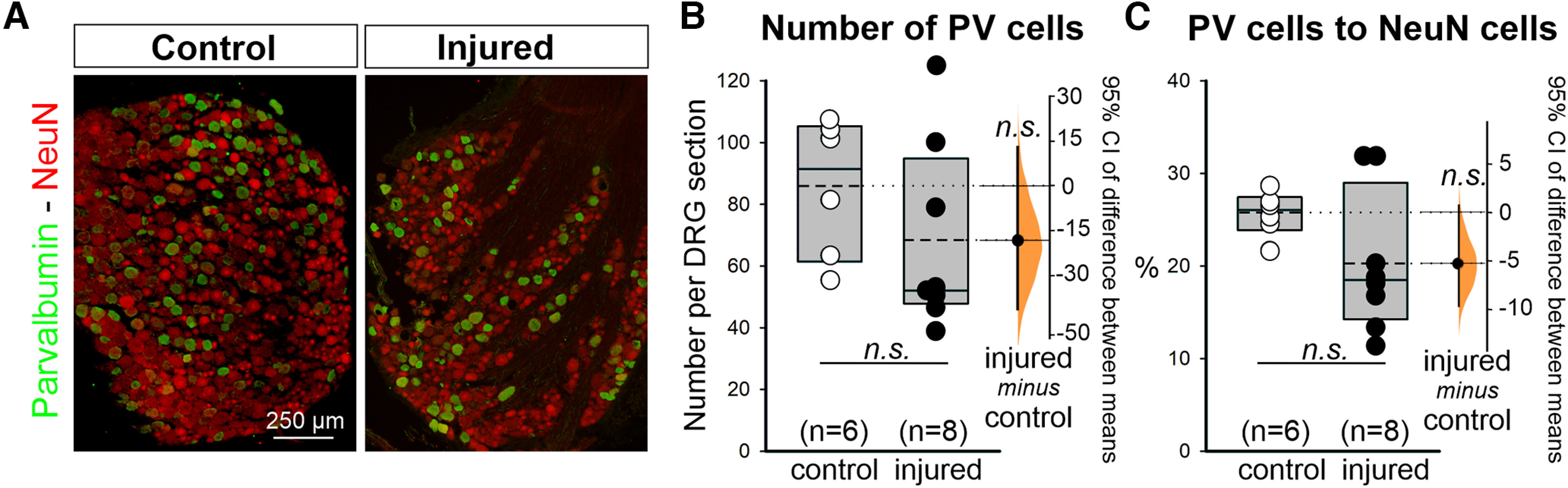
Lack of loss of proprioceptive neurons in DRG 60 d after crush injury. ***A***, Representative L4 DRG images of control (contralateral side) and injured (ipsilateral side) DRG neurons. PV immunofluorescence in green (FITC) and NeuN in red (Cy3). ***B***, Number of PV cells estimated by counting all parvalbumin in serial sections of L4 DRG and obtaining estimates per ganglia (each dot represents one DRG). Box plots represent the 25th to 75th percentile; the continuous line represents the median; the dashed line represents the average; *n* indicates the number of DRGs sampled. Two-tailed *t* tests indicate that differences are not statistically significant (*p* = 0.262; Extended Data Fig. 6-1). Right, Gardner–Altman estimation plot showing the mean difference (−17.5) depicted as a dot, and the 95% confidence interval (CI; −39.4, 12.4) indicated by the ends of the vertical confidence interval (CI) of the difference between the means from 5000 bootstrap samples taken. The *p* value of the two-sided permutation *t* test is 0.265. ***C***, No differences were found between the percentage of PV neurons to all NeuN comparing ipsilateral and contralateral to the injury. Box plots and data points are represented as in ***B*** with 95% CI of mean differences represented to the right with an average value of 5.4% and 95% CI between −9.8 and 0.7). Two-tailed *t* tests indicate that differences are not statistically significant (*p* = 0.129; Extended Data Fig. 6-1; *p* = 0.131 for the two-sided permutation *t* test estimating mean differences values). Both tests in ***B*** and ***C*** were underpowered, and therefore the lack of statistical significance needs to be interpreted cautiously. However, any possible effect size is small and therefore does not explain the central loss of VGluT1 synapses.

10.1523/ENEURO.0436-22.2023.f5-6 Figure 5-6 Statistical table for changes in number of VGluT1 contacts with age in each dendrite compartment after injury. Download Figure 5-6, DOCX file.

### Proprioceptive neurons in L4 DRG 2 weeks after crush injury

One possible explanation for the differences between crush injuries at P10 compared with the adult would be a higher incidence of cell death among proprioceptors in younger animals. To evaluate whether crush injury at P10 affects the survival of proprioceptive neurons, we calculated the number of PV^+^ neurons, a marker of proprioceptive neurons (de Nooij et al., 2013; Oliver et al., 2021), and their percentage to all NeuN neurons in the right (injured side) and left (control) L4 DRGs 60 d after injury. We counted four to five sections per DRG and analyzed eight DRGs ipsilateral to the injury and six on the contralateral side. In the control side, we estimated 85.6 ± 22.4 (±SD) PV neurons per DRG 15-μm-thick section, and on the injured side, 68.2 ± 30.7. Similarly, we counted 299.7 ± 54.8 NeuN cells in control and 311.4 ± 68.9 in the injured side. The high variability is because of differences in the number of cells contained within each section depending on section area and cellular content. To avoid biases introduced by section level, we included in the quantitative analyses only sections with >200 NeuN neurons. The differences in PV cell numbers were not statistically significant (*t* tests; PV cells: *p* = 0.263, *t*_(12)_ = 1.175; NeuN cells: *p* = 0.738, *t*_(12)_ = 0.343). The percentage of PV cells to all NeuN cells was 25.7 ± 2.4% in control DRGs and 20.3 ± 7.2% in injured DRG sections. This percentage is similar to previous estimates reported in the literature (Patel et al., 2003), and the differences in DRGs ipsilateral and contralateral to the injury were not significant (*t* test: *p* = 0.129, *t*_(12)_ = 1.628). There was, however, large variability from DRG to DRG, and the powers of these tests for α = 0.05 were 0.296 (for PV cell numbers) and 0.323 (for PV percentage of NeuN cells), which are below the desired 0.8. However, after 5000 bootstrap sample permutations, the estimated effect size for the unpaired mean difference between control and axotomy is −17.5 (95.0% CI, −39.4, 12.4) for cell numbers and −5.35 (95.0% CI, −9.79, 0.68) for PV percentage versus NeuN with, respectively, *p* = 0.265 and *p* = 0.131 for the two-sided permutation *t* test (Fig. 6*B*,*C*). Therefore, conclusions derived from parametric mean testing and effect size statistics are in good agreement. Given the small sample size, a possible small significant effect in cell survival could still be detected by stronger powered statistics; however, the estimated effect size is too small to explain the large changes (depletions, 70–40%) in VGluT1 synaptic coverage on MNs. Injury-induced cell death of PV^+^ DRG neurons thus cannot explain deficits of VGluT1 contacts on α-motoneurons after nerve crush injury at P10.

10.1523/ENEURO.0436-22.2023.f6-1 Figure 6-1 Statistical table for changes in number of PV cells in the L4 DRG at 60 dpi. Download Figure 6-1, DOCX file.

### The central neuroinflammation reaction and microglia reactivity

**Figure 7.**
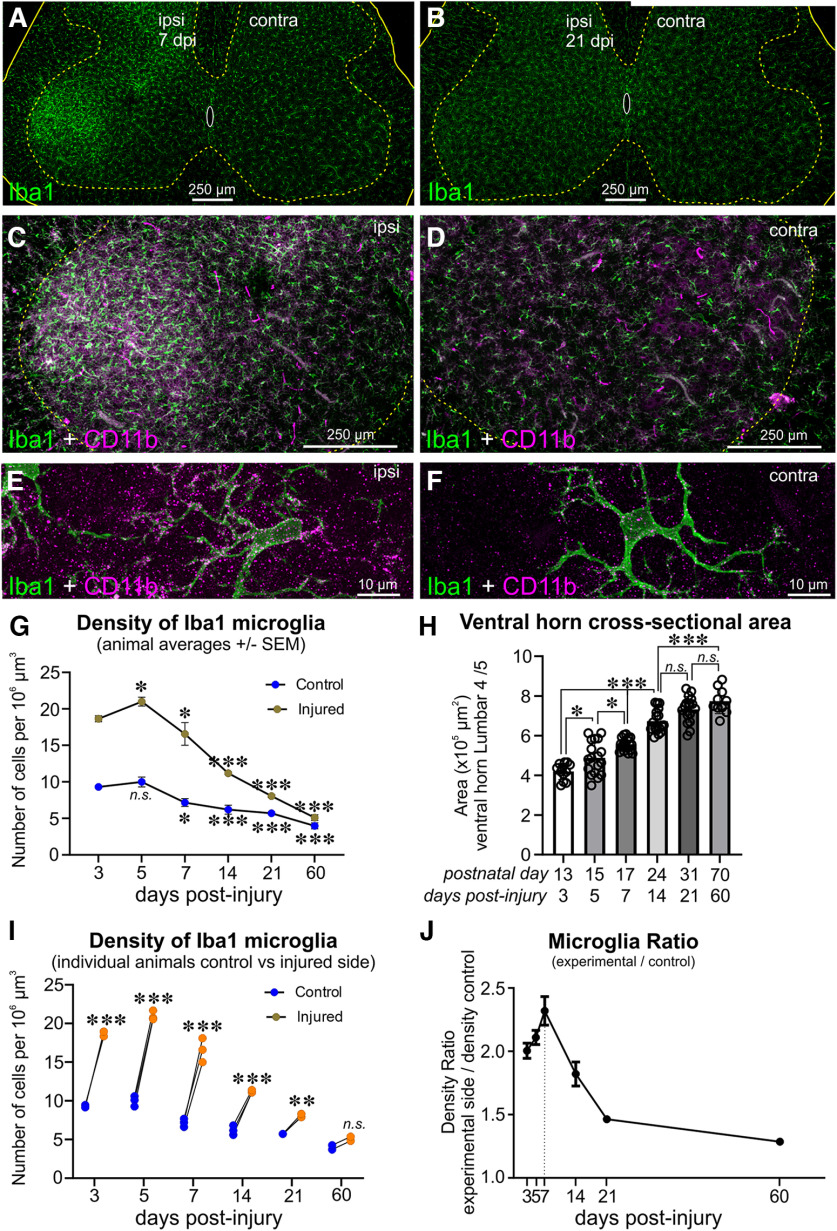
Microgliosis after sciatic nerve crush injury at P10. ***A***, ***B***, Representative images of Iba1-microglia in the spinal cords of animals at 7 dpi (***A***) and 21 dpi (***B***) after a unilateral nerve injury at P10. ***C***, ***D***, High-magnification images of ipsilateral (***C***) and contralateral (***D***) ventral horn Iba1 microglia (green) showing also immunoreactivity for C11b (blue). ***E***, ***F***, High-magnification images of Iba1-labeleld microglia (green) on the ipsilateral side (***E***) and the contralateral side (***F***) showing increased CD11b immunoreactivity (blue). ***G***, Iba1 microglia density in control and injured side. Asterisks show significance with respect to 3 d (*p < 0.05, **p < 0.01, ***p < 0.001; *post hoc* Bonferroni’s tests after two-way ANOVA for postnatal date and injury. Extended Data Figure 7-1; *n* = 3 animals at all ages except for 60 dpi in which *n* = 2). Density diminished in both the control and injured side with age because of spinal cord expansion. Density diminished faster and from a higher peak ipsilateral to the injury because of the additional induction and reversion of microgliosis. ***H***, Progressive growth of the area occupied by the ventral horn at different postnatal days (correspondence with days after injury are noted on the *x*-axis). Asterisks indicate significance, as above (*post hoc* Bonferroni’s tests after one-way ANOVA for postnatal date; Extended Data Fig. 7-2). ***I***, Quantitative comparison of microglia cell numbers ipsilateral versus contralateral to the injury at each time point. Asterisks indicate significance as in ***H*** (*post hoc* Bonferroni’s tests after the same two-way ANOVA as in ***H***; Extended Data Fig. 7-1). ***J***, Microglia density ratios comparing the side ipsilateral to the injury to the contralateral side (ratio, average ± SEM). The largest increase in microgliosis, considering the effect of changing spinal cord sizes, occurs at 7 d and then rapidly decreases.

The microglial reaction in the lumbar spinal cord was analyzed at 3, 5, 7, 14, and 21 dpi after a P10 injury in sections immunostained for Iba1 and CD11b (OX-42 antibody; Fig. 7*A*,*B*). These markers are also shared by infiltrating immune cells and therefore serial sections were labeled with CD45 antibodies to detect any CD45^high^ blood-derived inflammatory cells. We could not detect any CD45^high^ cells in our samples at any age, and therefore our quantitative analyses focused on Iba1/CD11b cells likely derived from resident microglia. We counted microglia in the ipsilateral (injured) and contralateral (control) gray matter of the ventral horn and normalized the counts to the available volume (area of the ventral horn × section thickness). We used three animals per time point from 5 to 21 dpi and two animals at 3 dpi. We imaged three sections per animal comparing both sides of the cord to obtain one estimate per animal (Fig. 7*C*,*D*). In the side ipsilateral to the injury not only microglia number increased, but also Iba1 and CD11b immunoreactivities (Fig. 7*C–F*). The density of Iba1 microglia in the injured ventral horn was significantly higher than in the control side at all postinjury ages, but in addition there were also developmental changes in cell density with postnatal time because of the progressive increase in the size of the ventral horn (Fig. 7*G*). A two-way ANOVA for injury and time after injury (also including developmental time) showed significant differences according to both variables and a significant interaction (all *p* < 0.0001; Extended Data Fig. 7-1). The density of microglia peaked at 5 dpi, corresponding to P15, but while density was significantly increased at 5 dpi compared with 3 dpi in the injured side (*p* < 0.05, *post hoc* Bonferroni tests; Fig. 7*C*), the differences were not significant in the control side (*p* > 0.999). Thereafter, density was significantly reduced in both sides, most likely because of progressive significant increases in ventral horn size with age (Fig. 7*H*). Nevertheless, the differences in cell density between injured and uninjured sides remained significant at all time points analyzed (Fig. 7*I*). When microglia density on the injured side was calculated as a ratio of the density on the control side (normalizing for changes in density because of development), maximal microgliosis was detected at 7 dpi. At 3 dpi, there was already microgliosis (ratio, 2.01 ± 0.06), increased at 5 dpi (2.11 ± 0.06), peaked at 7 dpi (2.32 ± 0.11), and decreased afterward (14 dpi, 1.82 ± 0.1; 21 dpi, 1.46 ± 0.01; Fig. 7*J*). Microglia density returned toward control values slowly, and there were still significantly more microglia ipsilateral to the injury at 21 d postinjury. Two animals were further processed at 60 dpi, and in these animals Ib1a/CD11b microglia appearance and density were almost identical on both sides.

10.1523/ENEURO.0436-22.2023.f7-1 Figure 7-1 Statistical table for changes in microglia number after nerve crush injury at P10. Download Figure 7-1, DOCX file.

10.1523/ENEURO.0436-22.2023.f7-2 Figure 7-2 Statistical table for changes in ventral horn size with postnatal developmental maturation. Download Figure 7-2, DOCX file.

Thus, activation of microglia after P10 nerve crush injury included rapid proliferation and upregulation of complement receptor components, followed by fast downregulation in the second week after injury and no infiltration of peripheral immune cells. This time course is faster than previously shown in mouse after nerve transection in the adult and suggests that the permanent deletion of VGluT1 synapses after P10 nerve crush involves a microglia mechanism during the first week after injury by difference to the neuroinflammation mechanisms described previously in the second week after adult nerve transection (Rotterman et al., 2019).

## Discussion

In this article, we show that a crush nerve injury in the early postnatal period (P10) induces a massive loss of VGluT1 synapses on spinal α-motoneurons by 60 d postinjury, despite almost complete muscle reinnervation at this time. These VGluT1 synapses on cell bodies and proximal dendrites of LG α-motoneurons are interpreted as being derived from muscle spindle proprioceptors and not corticospinal axons (another source of VGluT1 synapses in the lumbar ventral horn; Alvarez et al., 2004; Persson et al., 2006; Du Beau et al., 2012) because the corticospinal tract does not directly innervate motoneurons in the lateral motor columns of lumbar segments in normal mice and any cortico–motoneuronal connections formed during early development are efficiently pruned later (Bareyre et al., 2005; Du Beau et al., 2012; Gu et al., 2017; Moreno-Lopez et al., 2021). Within sensory afferent VGluT1 synapses, those in synaptic contact with α-motoneurons could belong to either type Ia or type II spindle afferents, but not to Golgi tendon organ Ib afferents or cutaneous mechanoreceptors that do not enter the ventral horn (Alvarez et al., 2011; Vincent et al., 2017). Ia and II ventrally synaptic collaterals reach Lamina IX to innervate α-motoneurons in the rodent spinal cord (Vincent et al., 2017), but the relative abundance of synapses from each type of sensory axon suggest a strong bias toward Ia identity.

The large loss of VGluT1/Ia/II synapses on spinal α-motoneurons by 60 d postinjury is clearly different from the response to nerve crush in adult rats, in which there is a significant 35–38% decrease in VGluT1 synaptic density on the cell bodies but no significant decrease on the dendrites by 21 d or 3 months after nerve injuries (Schultz et al., 2017). In contrast, we detected a larger depletion of VGluT1 synaptic densities on cell bodies (>60%) and dendrites (∼50% at all distance bins) at 60 d postinjury when the nerve crush occurs at P10. This loss was comparable to that detected in adult rats after nerve transections (Alvarez et al., 2011). We targeted the sciatic nerve at mid-thigh position for neonatal nerve injuries and for the tibial nerve after the sciatic nerve trifurcation for adult injuries. We believe that results among these different injuries are comparable since analyses are focused on LG MNs retrogradely labeled before the injury. The LG MN pool receives monosynaptic inputs from Ia afferents, and to a lesser extent II afferents, innervating all triceps surae muscles, and these travel in both the sciatic and tibial nerves. We have shown previously that the permanent removal of VGluT1/Ia/II afferent synapses from MNs is dependent on injury in the peripheral nerve to the Ia afferent, and not to the MN (Alvarez et al., 2011; Rotterman et al., 2019; Alvarez et al., 2020). In other words, Ia/II afferents retract from ventral horn targets, including interneurons, injured MNs, and uninjured MNs, independent of the injury status of the postsynaptic neuron. Ia/II afferents presynaptic to LG MNs are similarly injured by nerve crush at the sciatic or tibial nerve level, and their response is expected to be independent of the size of the injured MN cohort. Thus, the difference between the present results and those previously reported in the adult rat after nerve crush injuries (Schultz et al., 2017) requires an alternative explanation.

One major difference is developmental stage. It is well known that during the first 2 weeks of postnatal life in rodents, VGluT1/Ia/II synaptic densities progressively increase on their neuronal targets in the ventral horn (Mentis et al., 2006; Siembab et al., 2010; Mentis et al., 2011). In particular, VGluT1/Ia/II synapses on the cell body and dendrites of mouse MNs increase almost four times from P4 to P13 (Mentis et al., 2011). Here we found continued addition of synapses from P17 to P70, in part to match the increase in available surface with late postnatal development neuronal growth. In a detailed previous study (Kalb, 1994), it was shown that during the third postnatal week rat MNs exhibit significant growth in cell body size (confirmed in our study) and also dendritic length and bifurcations. The effects of sciatic nerve crush were detectable in both a loss of synapses and a slowing of synaptic accumulations during this period of MN growth, perhaps suggesting that remaining additions occur in inputs of decreased strength with less proliferative capacity. Together, they result in a large reduction in synaptic inputs from VGluT1/Ia/II afferents on the mature MN after it has regenerated and reinnervated muscle.

One possible explanation for the above results is that either MNs or Ia/II afferents are more susceptible to dying after injury to their axons in young animals. Alternatively, we previously demonstrated that the loss of VGluT1 Ia/II synapses in the ventral horn is dependent on a microglia mechanism and the ventral horn neuroinflammation in response to the injury in the peripheral nerve (Rotterman et al., 2019). Given that the neuroinflammatory reaction in the dorsal horn in response to nerve injuries is different in neonates, young rats, and adult rats (Vega-Avelaira et al., 2007), we sought to compare the time course and properties of the microglia reaction in the ventral horn of P10 injured animals with previous observations after similar injuries in adult. We hypothesized that a biased microglia phenotype at this age toward synaptic pruning might induce a differential effect on Ia/II synapse preservation when combined with further activation because of peripheral nerve injuries. This possibility is further supported by a recent report demonstrating microglia synaptic pruning of Ia afferent synapses in the neonate via a canonical C1q complement mechanism (Vukojicic et al., 2019).

### Cell death cannot explain the massive loss of VGluT1 synapses

We applied nerve crush injuries in P10 rats because motoneuron and sensory DRG cell death is expected to be limited at this age (Lowrie and Vrbová, 1992; Kemp et al., 2015). A direct comparison of MN and DRG cell survival after sciatic nerve crush in P3, P5, P7, P30, and adult rats showed that, while injury at P3 resulted in loss of MNs and DRGs in >50% of cells, these losses were minimal (<20%) when the injury occurred at P7, and limited cell loss was only observed in the DRGs when injuries were conducted at P30 (Kemp et al., 2015). In our study, we estimated that CMAP amplitudes would recover to ∼70% of contralateral values 2 months after the injury. In agreement, histological analysis indicated almost full reinnervation of neuromuscular junctions at that time point. These data suggest that regeneration after P10 sciatic crush results in muscle innervation close to normal. This is expected because, in the absence of motoneuron loss, surviving axons in the proximal stump after nerve crush injuries are effectively guided through endoneurial tubes that remain connected to the original peripheral targets facilitating reinnervation speed and specificity (Nguyen et al., 2002; Valero-Cabré et al., 2004).

Motoneuron sizes were different at P70 compared with previous ages. This could be because of the selective loss of smaller α-motoneurons or alternatively by biases introduced by the different tracers used in short-term (CTb-555) or long-term (Fast Blue) experiments. We believe, however, that the increase in cell body size is a normal developmental feature and cannot be explained by either of these alternative explanations. First, the coefficient of variation in size remained constant, suggesting spread in size range parallel to the mean increase. This is what is expected by the maturation of different sizes motor units and is similar to the increase in cell sizes of lumbar α-motoneurons that we reported before in mice (Shneider et al., 2009) and others in the rat (Westerga and Gramsbergen, 1992; Ishihara et al., 1994). If the type of tracer or cell death had introduced a bias toward certain cell sizes, we should observe a scaling factor for the whole-cell size distribution, not an increase in range. Moreover, the increase in VGluT1 numbers seems to match the cell size increase, maintaining a constant synaptic density. Finally, motoneuron cell death after certain injuries in the mouse are accompanied by specific local microglia reactions merging in tight macroclusters around the degenerating motoneuron (Rotterman and Alvarez, 2020). These were never observed in our preparations.

Alternatively, a proportion of Ia/II afferent sensory neurons might die when disconnected from muscle at this developmental time point. Approximately 50% of sensory neurons die within 2–3 d after axotomy because of sciatic nerve transection or crush in P0 to P1 rat pups (Yip et al., 1984; Whiteside et al., 1998). In contrast, sciatic nerve crush in 5-week-old rats results in limited and protracted (occurring for several days) cell death of sensory neurons (Ekstrom, 1995). A recent study suggested that DRG neuron cell loss can occur after injuries during the juvenile age (Kemp et al., 2015). To address the possibility of some cell loss from proprioceptors after axotomy following the crush nerve injury at P10, we evaluated the percentage of PV^+^ neurons in DRGs 2 weeks after the injury. Parvalbumin is a well known marker of proprioceptive afferents (de Nooij et al., 2013; Oliver et al., 2021). We found a very small and nonsignificant reduction observed in the total number of PV^+^ cells or the ratio of PV cells to all NeuN DRG neurons. If the small change were to become significant by increasing sample size (number of animals), the estimated effect size would remain too small to explain the large loss of VGluT1/Ia/II synapses on MNs. Previous studies already suggested that large-diameter cutaneous and muscle afferents are more resistant to axotomy than small cutaneous afferents (Tandrup et al., 2000; Hu and McLachlan, 2003).

### Neuroinflammation after neonatal nerve crush differs from crush in the adult

Comparison of microglia in nerve cut versus crush in the adult rat showed that microglia activation is similar during the first 2 weeks postinjury but differs at later time points because of a prolongation of microglia activation after nerve cut (He et al., 1996; Campos et al., 2021). We obtained similar results in adult mice (Rotterman et al., 2019), and indeed the prolonged microglia activation correlated with infiltration of blood-borne CCR2 cells after nerve transection, but not after nerve crush, and the time course of immune cell infiltration correlated with the disappearance of VGluT1 synapses from the ventral horn (Rotterman et al., 2019). Moreover, VGluT1/Ia/II synapses were partially preserved in the cell bodies and fully preserved on the dendrites of MNs that regenerate and reinnervate muscle after axotomy in CCR2 knock-out animals (Rotterman et al., 2019). These results lead to the conclusion that a different neuroinflammatory environment after each type of injury, particularly at later time points after injury (>15 d), contributed to the permanent deletion of VGluT1/Ia/II afferent synapses. The time course of microglia activation following nerve crush injuries in the neonate has not been analyzed in detail before. Surprisingly, microgliosis declined quite fast after the first week of activation and despite a massive loss of VGluT1 synapses. Moreover, CD45 immunoreaction could not demonstrate any infiltration of peripheral immune cells at early or late postnatal ages. We conclude that the microglia reaction after crush injury at P10 differs from those in transection injuries in the adult and that the lack of CD45 cell infiltration suggests limited activation of CCR2 signaling. We therefore propose that the mechanism of loss of VGluT1/Ia/II synapses after nerve crush injury at P10 likely differs from mechanisms driving the loss of VGUT1/Ia/II synapses in the adult after nerve transection injuries.

Recent single-cell RNA sequencing analyses showed that microglia during early postnatal development display phenotypes highly active in synaptic and axon pruning (Matcovitch-Natan et al., 2016; Hammond et al., 2019). It is also known that Ia afferent synapses are actively pruned through C1q complement mechanisms during a critical window of synaptic selection and strengthening during the first 2 postnatal weeks (Vukojicic et al., 2019). Indeed, we found a large increase in CD11b expression in activated microglia. CD11b and CD18 to form CR3, a phagocytic complement receptor that activates downstream of C1q and contributes to synaptic plasticity during developmental synaptic pruning (Stephan et al., 2012). Therefore, it is possible that VGluT1/Ia/II synapse susceptibility to loss is enhanced when nerve crush injuries occur at postnatal stages when circuitries are undergoing the final stages of wiring plasticity using complement mechanisms and compared with similar injuries in adults in which we observed limited VGluT1/Ia/II plasticity (Schultz et al., 2017).

One question is whether microglia recognize Ia/II afferent synapses related to axotomized motoneurons or all ventral horn synapses from Ia/II afferents injured in the periphery, regardless of spinal postsynaptic targets. Indeed, the microglia reaction in the ventral horn distributes along the regions targeted by synapses from injured Ia/II proprioceptors, from medial lamina V to lateral lamina IX, crossing through LVII in the ventrolateral direction (Fig. 7*A*). Previously, we showed that after adult injuries in the mouse the microglia reaction in the ventral horn was critical, while dorsal horn microglia activation (induced by signals from sensory afferents) was dispensable (Rotterman et al., 2019). In the adult rat injury to one muscle nerve [e.g., the medial G (MG)] deletes Ia inputs from injured MG α-motoneurons, but also affects reciprocal inhibition of uninjured synergists (Horstman et al., 2019). This agrees with the die-back of ventrally directed Ia synaptic collaterals after nerve injury (Alvarez et al., 2011), suggesting retraction from not only axotomized α-motoneurons, but also other ventral horn targets, including uninjured synergists and ventral proprioceptive interneurons. Whether a similar die-back occurs after nerve crush in young animals will need to be assessed in the future by labeling individual regenerated Ia afferents, as before (Alvarez et al., 2011), and using more focalized nerve injuries to study homonymous and heteronymous connections.

### Functional implications

Deleterious effects on motor function after injuries in the postnatal period have been described even when there is no motoneuron death (Lowrie et al., 1982; Lowrie and Vrbová, 1984; Lowrie et al., 1987). For example, muscle tension recovers to only half of normal following muscle reinnervation after nerve crush injuries at P11 to P12 (Lowrie et al., 1987). Nerve crush injuries inflicted up to P30 caused permanent muscle impairments and did not recover full function (Kemp et al., 2015). These studies suggest that factors unrelated to motoneuron death lead to poor motor function recovery after crush injuries in the postnatal period. Ia afferent input to the ventral horn is very significant for motor function, from maintaining muscle tone to regulating motor unit recruitment during motor activity and serving for rapid online muscle adjustments during performance. Therefore, it is quite possible that the massive loss of VGluT1 synapses we report here could impair the recovery of motor function when nerve crush occurs in the neonate.

In conclusion, after crush injury in the postnatal stage, there is a major loss of Ia synapses on α-motoneurons. Further research should focus on identifying the mechanisms behind this permanent synaptic loss to improve the functional recovery after postnatal nerve injuries.

## Synthesis

Reviewing Editor: Jeffery Twiss, University of South Carolina Decisions are customarily a result of the Reviewing Editor and the peer reviewers coming together and discussing their recommendations until a consensus is reached. When revisions are invited, a fact-based synthesis statement explaining their decision and outlining what is needed to prepare a revision will be listed below. The following reviewer(s) agreed to reveal their identity: Leif havton, Jennifer Wilhelm.

This study provides experimental data and findings that support a previously less understood mechanism for age-dependent recovery after peripheral nerve injury. The authors report potential contributor to limited functional recovery after peripheral nerve injury. Specifically, they report an age-dependent effect of synaptic loss on motoneurons in a rat model that is more pronounced during specific age intervals, specifically providing information about how younger animals respond to nerve crush injury and how the responses compare to previously reported findings in adults.

Contributions by age-dependent microglia reactions are suggested. These responses to nerve crush injury have not been described in young rats before. So the reviewers noted that this gives insight into how different mechanisms may be used in younger animals compared to older animals. Overall, the study is overall well done technically and with good design, and both reviewers and the reviewing editor are enthusiastic over this submission. There are some issues that we feel addressing would significantly strengthen the manuscript - these likely can be addressed with text modifications and the reviewers wondered whether the authors would have data in hand to address major point # 4 below.

Major points:

1. The authors use two different tracers for retrograde labeling of motor neurons, CTb and FB. What was the rationale for using two separate tracers instead of a single tracer that could label motor neurons in both short-term and long-term studies? The issues may be of particular relevance, as the motoneurons labeled with different tracers were of different size. Could tracer choice have contributed to this difference in motor neuron size? Do the authors have any data on differential loss of axotomized motoneurons between the study groups that may have affected the size differences?

2. The authors excluded motoneurons with a cell body diameter less than 25 μm with an attempt to exclude gamma-motoneurons, which lack VGLUT1 inputs. This should be clearer in the Introduction, Discussion, and summary statements, that the study therefore is focused on the population of presumed alpha-motoneurons rather than the full population of “motoneurons”.

3. The authors use VGLUT1 as a marker for Ia afferents. However, VGLUT1 is also present in the corticospinal tract in the spinal cord (Persson et al., 2006, 2006, 497: 683-701). Could a CST source of VGLUT1 have influenced the results and interpretation of the study?

4. The age-dependent loss of VGLUT1 afferents on motoneurons in the rat spinal cord raises the question of whether this pattern of synaptic loss is specific for VLGUT1 afferents or more generally affects synaptic inputs to the motoneurons? Synaptophysin or other presynaptic-labeling may address this issue.

5. The authors should discuss whether the loss of synaptic input following a nerve crush injury is secondary to a motoneuron retrograde reaction to axonal injury or secondary to injury to primary afferents, or possibly a combination of these possible mechanisms.

Minor points:

1. The figure legend for Figure 7 does not contain a description of all of the images/graphs shown. The current legend refers to A-C, but those labels don’t match up with the data shown. Please update the figure to reflect the data shown in parts A-J.

2. Figure legend for Figure 2 should be updated to include a description of the difference between “reinnervated” and “fully reinnervated” rather than simply referring the reader to the extended data (which doesn’t explain the difference). The description of the two groups is fine in the body of the paper, but it creates confusion to not have some information about the groups represented in the figure legend, especially when the other figure legends are so descriptive.

3. In first paragraph of Discussion, a “non-significant decrease....” is not a decrease. Rewording of the sentence is suggested.

## Author Response

We thank the reviewers and the reviewing editor for their support and their helpful comments. We hope we have now clarified all the points that were raised and improved our manuscript. Estimation statistics were used when appropriate (see Figure 6). All changes are highlighted in yellow.

Major points:

1. The authors use two different tracers for retrograde labeling of motor neurons, CTb and FB. What was the rationale for using two separate tracers instead of a single tracer that could label motor neurons in both short-term and long-term studies? The issues may be of particular relevance, as the motoneurons labeled with different tracers were of different size. Could tracer choice have contributed to this difference in motor neuron size? Do the authors have any data on differential loss of axotomized motoneurons between the study groups that may have affected the size differences?

The choice of tracer depends on their stability inside the motoneurons. We retrogradely traced LG motoneurons before the injury, now we more clearly specified that it was done 2-3 days before. Thereafter there are different time points from 7 to 60 days post-injury. CTb is a protein and it is slowly degraded by the motoneurons. 14 days after muscle injection there is significantly decreased labeling and by 21 days almost none. Fast Blue on the other hand is stable for long periods of time inside the motoneurons (in our hands up to 6 months after the injection). Fast Blue experiments are significantly more expensive because a 10x higher concentration is required, so this tracer was used only for long-term studies.

We do not believe tracer choice affected the results: 1) the CV of sizes remained constant suggesting and spreading of motoneuron cell body sizes as should be expected with maturation of motor units of different sizes. If tracer was influencing the results, we should have seen a compression of CV due to the introduction of scaling factors related to tracer. This was not seen. 2) The increase in VGLUT1 synapses perfectly matches the increases in cell body size to maintain synaptic densities constant. It would have been very surprising if VGLUT1 synapse numbers were matching cell body sizes according to the tracer used. 3) In several unpublished data sets we retrace motoneurons after reinnervation. In this case motoneurons are labeled with Fast Blue before the injury and with CTb555 after reinnervation. In these experiments we do not see any difference in cell sizes between CTb555 and Fast Blue labeling. We do not refer to this study because is currently unpublished, but a manuscript including these data is being prepared for submission in early 2023. 4) This developmental increase and spreading in cell body sizes is identical to the one we reported in a previous study using genetic labeling of different motoneuron subtypes in mice (Schneider et al., 2009). It is also similar to what has been routinely reported in different motor pools of the cat and rat (two new references for the rat are now provided: Ishiara et al., 1992; Westerga and Gramsbergen, 1990).

We did not analyze motoneuron cell loss in this study, but relied on studies already published in the rat with the same nerve injuries we utilized here and suggesting there is no cell loss after nerve crush in animals older than P10. In other studies, we analyze motoneuron cell loss after nerve injury in mice, a species in which there is significant motoneuron cell death after many different types of injuries. Motoneuron cells loss is characterized by the formation of macroclusters of microglia and T-cells surrounding dying motoneurons (Rotterman et al., 2020). We did not observe these macroclusters at any time point analyzed.

We added a paragraph in the discussion section related to cell death to clarify all these points.

2. The authors excluded motoneurons with a cell body diameter less than 25 μm with an attempt to exclude gamma-motoneurons, which lack VGLUT1 inputs. This should be clearer in the Introduction, Discussion, and summary statements, that the study therefore is focused on the population of presumed alpha-motoneurons rather than the full population of “motoneurons”.

Thank you for pointing this out. We introduced changes in the introduction, discussion, and summary statements to make this clearer.

3. The authors use VGLUT1 as a marker for Ia afferents. However, VGLUT1 is also present in the corticospinal tract in the spinal cord (Persson et al., 2006, 2006, 497: 683-701). Could a CST source of VGLUT1 have influenced the results and interpretation of the study?

Lumbar mouse motoneurons and in particular, triceps surae motoneurons, do not receive monosynaptic inputs from the corticospinal tract on the proximal somatodendritic regions studied here. However, some VGLUT1 synapses could belong to type II afferents (the secondary sensory afferents in muscle spindles). We have added the following text to the start of the discussion and changed Ia for Ia/II throughout the text to increase accuracy.

In this paper we show that a crush nerve injury in the early postnatal period (p10) induces a massive loss of VGLUT1 synapses on spinal α-motoneurons by 60 days postinjury, despite almost complete muscle reinnervation at this time. These VGLUT1 synapses on cell bodies and proximal dendrites of LG α-motoneurons are interpreted as derived from muscle spindle proprioceptors and not corticospinal axons (another source of VGLUT1 synapses in the lumbar ventral horn; Alvarez et al., 2004; Persson et al., 2006; Du Beau et al., 2017) because the corticospinal tract does not directly innervate motoneurons in the lateral motor columns of lumbar segments in normal mice and any cortico-motoneuronal connections formed during early development are efficiently pruned later (Du Beau et al., 2017; Bareyre et al., 2005; Gu et al. 2017; Moreno-Lopez et al., 2021). Within sensory afferent VGLUT1 synapses, those in synaptic contact with α-motoneurons could belong to either type Ia or type II spindle afferents, but not to Golgi tendon organ Ib afferents or cutaneous mechanoreceptors that do not enter the ventral horn (Alvarez et al., 2011; Vincent et al., 2017). Ia and II ventrally synaptic collaterals reach Lamina IX to innervate α-motoneurons in the rodent spinal cord (Vincent et al., 2017), but the relative abundance of synapses from each type of sensory axon suggest a strong bias towards Ia identity.

4. The age-dependent loss of VGLUT1 afferents on motoneurons in the rat spinal cord raises the question of whether this pattern of synaptic loss is specific for VLGUT1 afferents or more generally affects synaptic inputs to the motoneurons? Synaptophysin or other presynaptic-labeling may address this issue.

This is a very important point. We agree that injuries early in postnatal development might induce large plasticity in spinal cord circuits that are network wide and distinct from adult synaptic and circuit plasticity. We did some synaptophysin labeling, but the results were unclear for technical issues (poor penetration of synaptophysin in tissue, very high densities in young spinal cords). Therefore, we prefer to address this question in future studies using more specific markers combined with genetic labeling for specific circuit elements The range of circuit changes that might occur in the young is yet a very sparsely studied issue in the spinal cord motor system.

5. The authors should discuss whether the loss of synaptic input following a nerve crush injury is secondary to a motoneuron retrograde reaction to axonal injury or secondary to injury to primary afferents, or possibly a combination of these possible mechanisms.

Because the loss of VGLUT1 synapses is not restricted to the axotomized motoneurons we believe that the injury of the primary afferent is the primary triggering event. This agrees with the observation that the die back of ventral horn Ia afferents collaterals affects the whole ventral horn and not only the pool of injured motoneurons (Alvarez et al., 2011). This necessarily denervates many targets in the ventral horn, either injured or uninjured by the nerve injury. The following text was added to the discussion.

One question is whether microglia recognize Ia/II afferent synapses related to axotomized motoneurons, or all ventral horn synapses from Ia/II afferents injured in the periphery, irrespective of spinal postsynaptic targets. Indeed, the microglia reaction in the ventral horn follows region targeted by synapses from injured Ia/II proprioceptors, from medial lamina V to lateral lamina IX, crossing through LVII in the ventro-lateral direction (Figure 7A). Previously, we showed that after adult injuries in the mouse the microglia reaction in the ventral horn was critical, while dorsal horn microglia activation (induced by signals from sensory afferents) was dispensable (Rotterman et al., 2019). In the adult rat injury to one muscle nerve (for example the medial gastrocnemius, MG) deletes Ia inputs from injured MG α-motoneurons, but also affects reciprocal inhibition of uninjured synergists (Hortsman et al., 2019). This agrees with the die-back of ventrally-directed Ia synaptic collaterals after nerve injury (Alvarez et al., 2011) suggesting retraction from not only axotomized α-motoneurons, but also other ventral horn targets, including uninjured synergists and ventral proprioceptive interneurons. Whether a similar die back occurs after nerve crush in young animals will need to be assess in the future by labeling individual regenerated Ia afferents, as before (Alvarez et al., 2011), and using more focalized nerve injuries to study homonymous and heteronymous connections.

Minor points:

1. The figure legend for Figure 7 does not contain a description of all of the images/graphs shown. The current legend refers to A-C, but those labels don’t match up with the data shown. Please update the figure to reflect the data shown in parts A-J.

Thank you for noting this! The figure legend was of a previous draft. A correct version is now included.

2. Figure legend for Figure 2 should be updated to include a description of the difference between “reinnervated” and “fully reinnervated” rather than simply referring the reader to the extended data (which doesn’t explain the difference). The description of the two groups is fine in the body of the paper, but it creates confusion to not have some information about the groups represented in the figure legend, especially when the other figure legends are so descriptive.

We expanded this Figure legend to explain the major points of the statistical comparisons. We prefer to omit the asterisks denoting significance from the figure because the large number of comparisons will make it unreadable. These can be found in the Extended table.

3. In first paragraph of Discussion, a “non-significant decrease....” is not a decrease. Rewording of the sentence is suggested.

Corrected. Thank you.

## References

[B1] Abelew TA, Miller MD, Cope TC, Nichols TR (2000) Local loss of proprioception results in disruption of interjoint coordination during locomotion in the cat. J Neurophysiol 84:2709–2714. 10.1152/jn.2000.84.5.2709 11068014

[B2] Altman J, Bayer SA (2001) Development of the human spinal cord. New York: Oxford UP.

[B3] Altman J, Sudarshan K (1975) Postnatal development of locomotion in the laboratory rat. Anim Behav 23:896–920. 10.1016/0003-3472(75)90114-1 1200422

[B4] Alvarez FJ, Villalba RM, Zerda R, Schneider SP (2004) Vesicular glutamate transporters in the spinal cord, with special reference to sensory primary afferent synapses. J Comp Neurol 472:257–280. 10.1002/cne.20012 15065123

[B5] Alvarez FJ, Bullinger KL, Titus HE, Nardelli P, Cope TC (2010) Permanent reorganization of Ia afferent synapses on motoneurons after peripheral nerve injuries. Ann N Y Acad Sci 1198:231–241. 10.1111/j.1749-6632.2010.05459.x 20536938 PMC2997487

[B6] Alvarez FJ, Titus-Mitchell HE, Bullinger KL, Kraszpulski M, Nardelli P, Cope TC (2011) Permanent central synaptic disconnection of proprioceptors after nerve injury and regeneration. I. Loss of VGLUT1/IA synapses on motoneurons. J Neurophysiol 106:2450–2470. 10.1152/jn.01095.2010 21832035 PMC3214094

[B7] Alvarez FJ, Rotterman TM, Akhter ET, Lane AR, English AW, Cope TC (2020) Synaptic plasticity on motoneurons after axotomy: a necessary change in paradigm. Front Mol Neurosci 13:68. 32425754 10.3389/fnmol.2020.00068 PMC7203341

[B8] Barker D, Young JZ (1947) Recovery of stretch reflexes after nerve injury. Lancet 1:704–707. 10.1016/s0140-6736(47)91454-2 20241152

[B9] Bareyre FM, Kerschensteiner M, Misgeld T, Sanes JR (2005) Transgenic labeling of the corticospinal tract for monitoring axonal responses to spinal cord injury. Nat Med 11:1355–1360. 10.1038/nm1331 16286922

[B10] Bodine-Fowler SC, Meyer RS, Moskovitz A, Abrams R, Botte MJ (1997) Inaccurate projection of rat soleus motoneurons: a comparison of nerve repair techniques. Muscle Nerve 20:29–37. 10.1002/(SICI)1097-4598(199701)20:1<29::AID-MUS4>3.0.CO;2-J 8995580

[B11] Bolívar S, Navarro X, Udina E (2020) Schwann cell role in selectivity of nerve regeneration. Cells 9:2131. 10.3390/cells9092131 32962230 PMC7563640

[B12] Bontioti EN, Kanje M, Dahlin LB (2003) Regeneration and functional recovery in the upper extremity of rats after various types of nerve injuries. J Peripher Nerv Syst 8:159–168. 10.1046/j.1529-8027.2003.03023.x 12904237

[B13] Brushart TM, Mesulam MM (1980) Alteration in connections between muscle and anterior horn motoneurons after peripheral nerve repair. Science 208:603–605. 10.1126/science.7367884 7367884

[B14] Bullinger KL, Nardelli P, Pinter MJ, Alvarez FJ, Cope TC (2011) Permanent central synaptic disconnection of proprioceptors after nerve injury and regeneration. II. Loss of functional connectivity with motoneurons. J Neurophysiol 106:2471–2485. 10.1152/jn.01097.2010 21832030 PMC3214087

[B15] Campos RMP, Barbosa-Silva MC, Ribeiro-Resende VT (2021) Comparison of effect of crush or transection peripheral nerve lesion on lumbar spinal cord synaptic plasticity and microglial dynamics. IBRO Neurosci Rep 10:225–235. 10.1016/j.ibneur.2021.05.002 34179871 PMC8211924

[B16] Cope TC, Clark BD (1993) Motor-unit recruitment in self-reinnervated muscle. J Neurophysiol 70:1787–1796. 10.1152/jn.1993.70.5.1787 8294953

[B17] Cope TC, Bonasera SJ, Nichols TR (1994) Reinnervated muscles fail to produce stretch reflexes. J Neurophysiol 71:817–820. 10.1152/jn.1994.71.2.817 8176445

[B18] Du Beau A, Shakya Shrestha S, Bannatyne BA, Jalicy SM, Linnen S, Maxwell DJ (2012) Neurotransmitter phenotypes of descending systems in the rat lumbar spinal cord. Neuroscience 227:67–79. 10.1016/j.neuroscience.2012.09.037 23018001

[B19] de Nooij JC, Doobar S, Jessell TM (2013) Etv1 inactivation reveals proprioceptor subclasses that reflect the level of NT3 expression in muscle targets. Neuron 77:1055–1068. 10.1016/j.neuron.2013.01.015 23522042 PMC3763960

[B20] Ekstrom PA (1995) Neurones and glial cells of the mouse sciatic nerve undergo apoptosis after injury in vivo and in vitro. Neuroreport 6:1029–1032. 7632888 10.1097/00001756-199505090-00020

[B21] Gordon T (2016) Nerve regeneration: understanding biology and its influence on return of function after nerve transfers. Hand Clin 32:103–117. 10.1016/j.hcl.2015.12.001 27094884

[B22] Gordon T (2020) Peripheral nerve regeneration and muscle reinnervation. Int J Mol Sci 21:8652. 10.3390/ijms21228652 33212795 PMC7697710

[B23] Gordon T, Chan KM, Sulaiman OA, Udina E, Amirjani N, Brushart TM (2009) Accelerating axon growth to overcome limitations in functional recovery after peripheral nerve injury. Neurosurgery 65:A132–A144. 10.1227/01.NEU.0000335650.09473.D3 19927058

[B24] Greensmith L, Vrbová G (1992) Alterations of nerve-muscle interaction during postnatal development influence motoneurone survival in rats. Brain Res Dev Brain Res 69:125–131. 10.1016/0165-3806(92)90129-k 1424084

[B25] Gu Z, et al. (2017) Control of species-dependent cortico-motoneuronal connections underlying manual dexterity. Science 357:400–404. 10.1126/science.aan3721 28751609 PMC5774341

[B26] Haftel VK, Bichler EK, Wang QB, Prather JF, Pinter MJ, Cope TC (2005) Central suppression of regenerated proprioceptive afferents. J Neurosci 25:4733–4742. 10.1523/JNEUROSCI.4895-04.2005 15888649 PMC6724774

[B27] Hammond BP, Manek R, Kerr BJ, Macauley MS, Plemel JR (2021) Regulation of microglia population dynamics throughout development, health, and disease. Glia 69:2771–2797. 10.1002/glia.24047 34115410

[B28] Hammond TR, Dufort C, Dissing-Olesen L, Giera S, Young A, Wysoker A, Walker AJ, Gergits F, Segel M, Nemesh J, Marsh SE, Saunders A, Macosko E, Ginhoux F, Chen J, Franklin RJM, Piao X, McCarroll SA, Stevens B (2019) Single-cell RNA sequencing of microglia throughout the mouse lifespan and in the injured brain reveals complex cell-state changes. Immunity 50:253–271.e6. 10.1016/j.immuni.2018.11.004 30471926 PMC6655561

[B29] Hart AM, Terenghi G, Wiberg M (2008) Neuronal death after peripheral nerve injury and experimental strategies for neuroprotection. Neurol Res 30:999–1011. 10.1179/174313208X362479 19079974

[B30] He BP, Tay SS, Leong SK (1996) Macrophage and microglial cell response after common peroneal nerve cut and crush in C57BL/6J mice. Neurodegeneration 5:73–80. 10.1006/neur.1996.0010 8731385

[B31] Ho J, Tumkaya T, Aryal S, Choi H, Claridge-Chang A (2019) Moving beyond *P* values: data analysis with estimation graphics. Nat Methods 16:565–566. 10.1038/s41592-019-0470-3 31217592

[B32] Horstman G, Housley SN, Cope TC (2019) Dysregulation of mechanosensory circuits coordinating the actions of antagonist motor pools following peripheral nerve injury and muscle reinnervation. Exp Neurol 318:124–134. 10.1016/j.expneurol.2019.04.017 31039333 PMC6588415

[B33] Hu P, McLachlan EM (2003) Selective reactions of cutaneous and muscle afferent neurons to peripheral nerve transection in rats. J Neurosci 23:10559–10567. 10.1523/JNEUROSCI.23-33-10559.2003 14627640 PMC6740909

[B34] Ishihara A, Tsuzimoto H, Suzuki H, Kasuga N (1994) Postnatal changes in cell body size and oxidative enzyme activity of spinal motoneurons innervating the rat tibialis anterior muscle. Brain Res Dev Brain Res 83:28–34. 10.1016/0165-3806(94)90176-7 7697869

[B35] Kalb RG (1994) Regulation of motor neuron dendrite growth by NMDA receptor activation. Development 120:3063–3071. 10.1242/dev.120.11.3063 7720552

[B36] Kemp SW, Chiang CD, Liu EH, Wood MD, Willand MP, Gordon T, Borschel GH (2015) Characterization of neuronal death and functional deficits following nerve injury during the early postnatal developmental period in rats. Dev Neurosci 37:66–77. 10.1159/000368769 25592862

[B37] Lowrie MB, Vrbová G (1984) Different pattern of recovery of fast and slow muscles following nerve injury in the rat. J Physiol 349:397–410. 10.1113/jphysiol.1984.sp015163 6737299 PMC1199344

[B38] Lowrie MB, Vrbová G (1992) Dependence of postnatal motoneurones on their targets: review and hypothesis. Trends Neurosci 15:80–84. 10.1016/0166-2236(92)90014-y 1373920

[B39] Lowrie MB, Krishnan S, Vrbová G (1982) Recovery of slow and fast muscles following nerve injury during early post-natal development in the rat. J Physiol 331:51–66. 10.1113/jphysiol.1982.sp014364 7153915 PMC1197741

[B40] Lowrie MB, Krishnan S, Vrbová G (1987) Permanent changes in muscle and motoneurones induced by nerve injury during a critical period of development of the rat. Brain Res 428:91–101. 10.1016/0165-3806(87)90086-1 3815121

[B41] Lundborg G (2000) A 25-year perspective of peripheral nerve surgery: evolving neuroscientific concepts and clinical significance. J Hand Surg Am 25:391–414. 10.1053/jhsu.2000.4165 10811744

[B42] Maas H, Prilutsky BI, Nichols TR, Gregor RJ (2007) The effects of self-reinnervation of cat medial and lateral gastrocnemius muscles on hindlimb kinematics in slope walking. Exp Brain Res 181:377–393. 10.1007/s00221-007-0938-8 17406860 PMC2712217

[B43] Madison RD, Robinson GA (2014) Accuracy of regenerating motor neurons: influence of diffusion in denervated nerve. Neuroscience 273:128–140. 10.1016/j.neuroscience.2014.05.016 24846614 PMC4096846

[B44] Madison RD, Archibald SJ, Brushart TM (1996) Reinnervation accuracy of the rat femoral nerve by motor and sensory neurons. J Neurosci 16:5698–5703. 10.1523/JNEUROSCI.16-18-05698.1996 8795625 PMC6578956

[B45] Matcovitch-Natan O, et al. (2016) Microglia development follows a stepwise program to regulate brain homeostasis. Science 353:aad8670. 10.1126/science.aad8670 27338705

[B46] Matthews PB (1981) Evolving views on the internal operation and functional role of the muscle spindle. J Physiol 320:1–30. 10.1113/jphysiol.1981.sp013931 6459449 PMC1244029

[B47] Mentis GZ, Siembab VC, Zerda R, O'Donovan MJ, Alvarez FJ (2006) Primary afferent synapses on developing and adult Renshaw cells. J Neurosci 26:13297–13310. 10.1523/jneurosci.2945-06.2006 17182780 PMC3008340

[B48] Mentis GZ, Blivis D, Liu W, Drobac E, Crowder ME, Kong L, Alvarez FJ, Sumner CJ, O'Donovan MJ (2011) Early functional impairment of sensory-motor connectivity in a mouse model of spinal muscular atrophy. Neuron 69:453–467. 10.1016/j.neuron.2010.12.032 21315257 PMC3044334

[B49] Molander C, Aldskogius H (1992) Directional specificity of regenerating primary sensory neurons after peripheral nerve crush or transection and epineurial suture A sequential double-labeling study in the rat. Restor Neurol Neurosci 4:339–344. 10.3233/RNN-1992-4505 21551665

[B50] Moreno-Lopez Y, Bichara C, Delbecq G, Isope P, Cordero-Erausquin M (2021) The corticospinal tract primarily modulates sensory inputs in the mouse lumbar cord. Elife 10:e65304. 10.7554/eLife.65304 34497004 PMC8439650

[B51] Navarro X, Verdú E, Buti M (1994) Comparison of regenerative and reinnervating capabilities of different functional types of nerve fibers. Exp Neurol 129:217–224. 10.1006/exnr.1994.1163 7957736

[B52] Navarro X, Vivó M, Valero-Cabre A (2007) Neural plasticity after peripheral nerve injury and regeneration. Prog Neurobiol 82:163–201. 10.1016/j.pneurobio.2007.06.005 17643733

[B53] Nguyen QT, Sanes JR, Lichtman JW (2002) Pre-existing pathways promote precise projection patterns. Nat Neurosci 5:861–867. 10.1038/nn905 12172551

[B54] Oliver KM, Florez-Paz DM, Badea TC, Mentis GZ, Menon V, de Nooij JC (2021) Molecular correlates of muscle spindle and Golgi tendon organ afferents. Nat Commun 12:1451. 10.1038/s41467-021-21880-3 33649316 PMC7977083

[B55] Patel TD, Kramer I, Kucera J, Niederkofler V, Jessell TM, Arber S, Snider WD (2003) Peripheral NT3 signaling is required for ETS protein expression and central patterning of proprioceptive sensory afferents. Neuron 38:403–416. 10.1016/s0896-6273(03)00261-7 12741988

[B56] Persson S, Boulland JL, Aspling M, Larsson M, Fremeau RT Jr, Edwards RH, Storm-Mathisen J, Chaudhry FA, Broman J (2006) Distribution of vesicular glutamate transporters 1 and 2 in the rat spinal cord, with a note on the spinocervical tract. J Comp Neurol 497:683–701. 10.1002/cne.20987 16786558

[B57] Prather JF, Nardelli P, Nakanishi ST, Ross KT, Nichols TR, Pinter MJ, Cope TC (2011) Recovery of proprioceptive feedback from nerve crush. J Physiol 589:4935–4947. 10.1113/jphysiol.2011.210518 21788349 PMC3224884

[B58] Rotterman TM, Alvarez FJ (2020) Microglia dynamics and interactions with motoneurons axotomized after nerve injuries revealed by two-photon imaging. Sci Rep 10:8648. 10.1038/s41598-020-65363-9 32457369 PMC7250868

[B59] Rotterman TM, Nardelli P, Cope TC, Alvarez FJ (2014) Normal distribution of VGLUT1 synapses on spinal motoneuron dendrites and their reorganization after nerve injury. J Neurosci 34:3475–3492. 10.1523/JNEUROSCI.4768-13.2014 24599449 PMC3942569

[B60] Rotterman TM, Akhter ET, Lane AR, MacPherson KP, Garcia VV, Tansey MG, Alvarez FJ (2019) Spinal motor circuit synaptic plasticity after peripheral nerve injury depends on microglia activation and a CCR2 mechanism. J Neurosci 39:3412–3433. 30833511 10.1523/JNEUROSCI.2945-17.2019 PMC6495126

[B61] Sabatier MJ, To BN, Nicolini J, English AW (2011a) Effect of slope and sciatic nerve injury on ankle muscle recruitment and hindlimb kinematics during walking in the rat. J Exp Biol 214:1007–1016. 10.1242/jeb.051508 21346129 PMC3044077

[B62] Sabatier MJ, To BN, Nicolini J, English AW (2011b) Effect of axon misdirection on recovery of electromyographic activity and kinematics after peripheral nerve injury. Cells Tissues Organs 193:298–309. 10.1159/000323677 21411964 PMC3128140

[B63] Schultz AJ, Rotterman TM, Dwarakanath A, Alvarez FJ (2017) VGLUT1 synapses and P-boutons on regenerating motoneurons after nerve crush. J Comp Neurol 525:2876–2889. 10.1002/cne.24244 28543879 PMC8063217

[B64] Shneider NA, Brown MN, Smith CA, Pickel J, Alvarez FJ (2009) Gamma motor neurons express distinct genetic markers at birth and require muscle spindle-derived GDNF for postnatal survival. Neural Dev 4:42. 10.1186/1749-8104-4-42 19954518 PMC2800842

[B65] Siembab VC, Smith CA, Zagoraiou L, Berrocal MC, Mentis GZ, Alvarez FJ (2010) Target selection of proprioceptive and motor axon synapses on neonatal V1-derived Ia inhibitory interneurons and Renshaw cells. J Comp Neurol 518:4675–4701. 10.1002/cne.22441 20963823 PMC3038681

[B66] Stephan AH, Barres BA, Stevens B (2012) The complement system: an unexpected role in synaptic pruning during development and disease. Annu Rev Neurosci 35:369–389. 10.1146/annurev-neuro-061010-113810 22715882

[B67] Tandrup T, Woolf CJ, Coggeshall RE (2000) Delayed loss of small dorsal root ganglion cells after transection of the rat sciatic nerve. J Comp Neurol 422:172–180. 10.1002/(SICI)1096-9861(20000626)422:2<172::AID-CNE2>3.0.CO;2-H 10842225

[B68] Valero-Cabré A, Navarro X (2001) H reflex restitution and facilitation after different types of peripheral nerve injury and repair. Brain Res 919:302–312. 10.1016/s0006-8993(01)03052-9 11701142

[B69] Valero-Cabré A, Tsironis K, Skouras E, Navarro X, Neiss WF (2004) Peripheral and spinal motor reorganization after nerve injury and repair. J Neurotrauma 21:95–108. 10.1089/089771504772695986 14987469

[B70] Vega-Avelaira D, Moss A, Fitzgerald M (2007) Age-related changes in the spinal cord microglial and astrocytic response profile to nerve injury. Brain Behav Immun 21:617–623. 10.1016/j.bbi.2006.10.007 17158026

[B71] Vincent JA, Gabriel HM, Deardorff AS, Nardelli P, Fyffe REW, Burkholder T, Cope TC (2017) Muscle proprioceptors in adult rat: mechanosensory signaling and synapse distribution in spinal cord. J Neurophysiol 118:2687–2701. 10.1152/jn.00497.2017 28814636 PMC5672542

[B72] Vukojicic A, Delestrée N, Fletcher EV, Pagiazitis JG, Sankaranarayanan S, Yednock TA, Barres BA, Mentis GZ (2019) The classical complement pathway mediates microglia-dependent remodeling of spinal motor circuits during development and in SMA. Cell Rep 29:3087–3100.e7. 10.1016/j.celrep.2019.11.013 31801075 PMC6937140

[B73] Westerga J, Gramsbergen A (1990) The development of locomotion in the rat. Brain Res Dev Brain Res 57:163–174. 10.1016/0165-3806(90)90042-w 2073717

[B74] Westerga J, Gramsbergen A (1992) Structural changes of the soleus and the tibialis anterior motoneuron pool during development in the rat. J Comp Neurol 319:406–416. 10.1002/cne.903190307 1602051

[B75] Whiteside G, Doyle CA, Hunt SP, Munglani R (1998) Differential time course of neuronal and glial apoptosis in neonatal rat dorsal root ganglia after sciatic nerve axotomy. Eur J Neurosci 10:3400–3408. 10.1046/j.1460-9568.1998.00346.x 9824453

[B76] Yip HK, Rich KM, Lampe PA, Johnson EM Jr (1984) The effects of nerve growth factor and its antiserum on the postnatal development and survival after injury of sensory neurons in rat dorsal root ganglia. J Neurosci 4:2986–2992. 10.1523/JNEUROSCI.04-12-02986.1984 6502217 PMC6564849

