# Supplementary material for: Massive Loss of Proprioceptive Ia Synapses in Rat Spinal Motoneurons after Nerve Crush Injuries in the Postnatal Period
Source: eNeuro. 2023 Feb 14;10(2):ENEURO.0436-22.2023. doi: 10.1523/ENEURO.0436-22.2023 (PMC9948128; doi:10.1523/ENEURO.0436-22.2023)
Supplement: Figure 1-1 — Statistical table for CMAP comparisons at different postinjury dates. Download Figure 1-1, DOCX file. [file enu-eN-NWR-0436-22-s03.docx]

**Extended data table Figure 1-1. Statistical table for CMAP comparisons at different postinjury dates.**

| **Tibialis Anterior (EMG CMAP data)**  i = ipsilateral to the injury; c = control contralateral to the injury  Two-way ANOVA for injury and days-post-injury (dpi)   - Injury: F_(69,1)_ = 324.33 p < 0.001 - dpi: F_(69,2)_ = 62.334 p < 0.001 - Injury X dpi: F_(69,2)_ = 20.314 p < 0.001 | | | | | | |
| --- | --- | --- | --- | --- | --- | --- |
| Groups | Mean c  mV ± SD | Mean i  mV ± SD | N  (animals) | Difference  Of Means | Adjusted p  Bonferroni | t |
| 14 dpi (p24) | 38.6 ± 7.2 | 2.5 ± 2.0 | 18 | 36.1 | <0.001*** | 20.826 |
| 21 dpi (p31) | 41.5 ± 7.0 | 13.3 ± 2.4 | 12 | 28.3 | <0.001*** | 13.319 |
| 60 dpi (p70) | 47.4 ± 1.4 | 34.6 ± 6.5 | 5 | 12.7 | <0.001*** | 3.869 |
| 14c vs 21c |  |  |  | 2.9 | 0.412 | 1.504 |
| 14c vs 60c |  |  |  | 8.8 | 0.004** | 3.344 |
| 21c vs 60c |  |  |  | 5.9 | 0.113 | 2.123 |
| 14i vs 21i |  |  |  | 10.7 | <0.001*** | 5.541 |
| 14i vs 60i |  |  |  | 32.2 | <0.001*** | 12.236 |
| 21i vs 60i |  |  |  | 21.4 | <0.001*** | 7.742 |
| **Gastrocnemius (EMG CMAP data)**  i = ipsilateral to the injury; c = control contralateral to the injury  Two-way ANOVA for injury and days-post-injury (dpi)   - Injury: F_(69,1)_ = 426.024 p < 0.001 - dpi: F_(69,2)_ = 153.600 p < 0.001 - Injury X dpi: F_(69,2)_ = 14.951 p < 0.001 | | | | | | |
| Groups | Mean c  mV ± SD | Mean i  mV ± SD | N  (animals) | Difference  Of Means | Adjusted p  Bonferroni | t |
| 14 dpi (p24) | 40.0 ± 6.7 | 4.0 ± 2.2 | 18 | 36.0 | <0.001*** | 22.489 |
| 21 dpi (p31) | 42.3 ± 3.1 | 16.8 ± 3.8 | 12 | 25.5 | <0.001*** | 13.040 |
| 60 dpi (p70) | 62.0 ± 8.1 | 42.0 ± 5.0 | 5 | 20.0 | <0.001*** | 6.589 |
| 14c vs 21c |  |  |  | 2.3 | 0.583 | 1.311 |
| 14c vs 60c |  |  |  | 22.1 | <0.001*** | 9.092 |
| 21c vs 60c |  |  |  | 19.7 | <0.001*** | 7.717 |
| 14i vs 21i |  |  |  | 12.8 | <0.001*** | 7.141 |
| 14i vs 60i |  |  |  | 38.0 | <0.001*** | 15.666 |
| 21i vs 60i |  |  |  | 25.2 | <0.001*** | 9.879 |
