# Supplementary material for: Massive Loss of Proprioceptive Ia Synapses in Rat Spinal Motoneurons after Nerve Crush Injuries in the Postnatal Period
Source: eNeuro. 2023 Feb 14;10(2):ENEURO.0436-22.2023. doi: 10.1523/ENEURO.0436-22.2023 (PMC9948128; doi:10.1523/ENEURO.0436-22.2023)
Supplement: Figure 2-1 — Statistical table for NMJ reinnervation at different postinjury dates. Download Figure 2-1, DOCX file. [file enu-eN-NWR-0436-22-s04.docx]

**Extended data table Figure 2-1. Statistical table for NMJ reinnervation at different postinjury dates.**

| **Tibialis Anterior Percentage of fully occupied NMJs**  (animal averages)  Normality, Shapiro-Wilk test: p=0.5543; passed normality tests (α = 0.05)  One-Way ANOVA for differences with days-post-injury (dpi)   - F_(3,7)_ = 49.10 p < 0.0001 | | | | | | |
| --- | --- | --- | --- | --- | --- | --- |
| Days after injury | Mean 1  % | Mean 2  % | n1 , n2  (animals) | 95% CI of  difference | Adjusted p  Bonferroni | t |
| 7 vs. 14 | 0.0 | 19.2 | 3 , 3 | -48.4 to 9.9 | 0.2859 | 2.397 |
| 7 vs 21 | 0.0 | 71.1 | 3 , 3 | -100.3 to -42.0 | <0.001*** | 8.876 |
| 7 vs 60 | 0.0 | 91.0 | 3 , 2 | -123.6 to -58.4 | <0.001*** | 10.16 |
| 14 vs 21 | 19.2 | 71.1 | 3 , 3 | -81.05 to -22.78 | 0.0020** | 6.478 |
| 14 vs 60 | 19.2 | 91.0 | 3 , 2 | -104.4 to -39.21 | <0.001*** | 8.012 |
| 21 vs 60 | 71.1 | 91.0 | 3 , 2 | -52.44 to 12.71 | 0.3727 | 2.217 |
| **Gastrocnemius Percentage of fully occupied NMJs**  (animal averages)  Normality, Shapiro-Wilk test: p=0.8460; passed normality tests (α = 0.05)  One-Way ANOVA for differences with days-post-injury (dpi)   - F_(3,7)_ = 20.00 p = 0.0008 | | | | | | |
| Days after injury | Mean 1  % | Mean 2  % | n1 , n2  (animals) | 95% CI of  difference | Adjusted p  Bonferroni | t |
| 7 vs. 14 | 0.0 | 45.3 | 3 , 3 | -88.1 to -2.6 | 0.0375* | 3.854 |
| 7 vs 21 | 0.0 | 71.3 | 3 , 3 | -114.1 to -28.6 | 0.0030** | 6.068 |
| 7 vs 60 | 0.0 | 93.3 | 3 , 2 | -141.1 to -45.6 | 0.0012** | 7.101 |
| 14 vs 21 | 45.3 | 71.3 | 3 , 3 | -68.8 to 16.7 | 0.3747 | 2.214 |
| 14 vs 60 | 45.3 | 93.3 | 3 , 2 | -95.8 to -0.2 | 0.0488* | 3.654 |
| 21 vs 60 | 71.3 | 93.3 | 3 , 2 | -69.8 to 25.8 | 0.8283 | 1.674 |
