# Supplementary material for: Massive Loss of Proprioceptive Ia Synapses in Rat Spinal Motoneurons after Nerve Crush Injuries in the Postnatal Period
Source: eNeuro. 2023 Feb 14;10(2):ENEURO.0436-22.2023. doi: 10.1523/ENEURO.0436-22.2023 (PMC9948128; doi:10.1523/ENEURO.0436-22.2023)
Supplement: Figure 3-1 — Statistical table for changes in cell body total surface with age. Download Figure 3-1, DOCX file. [file enu-eN-NWR-0436-22-s05.docx]

**Extended data table Figure 5-4. Statistical table for changes in dendrite VGLUT1 surface density according to age, injury and distance from the cell body.**

i = ipsilateral to injury; c = control contralateral to injury (pooled data per animal average)

| Normality, Shapiro-Wilk test: p > 0.2 in all data sets; pass normality test (α = 0.05)  Two-way ANOVA for dpi and distance in injury and control.   - dpi (control/injured): F_(2, 60)_ = 3.237 p = 0.0463 - dendritic compartment F_(5,60)_ = 29.26 p < 0.0001 - interaction: F_(10, 60)_ = 0.1208 p = 0.9995   Multiple comparisons Bonferroni corrected t-tests | | | | | | |
| --- | --- | --- | --- | --- | --- | --- |
| **VGLUT1 surface density p17** | | | | | | |
| Dendrite bins  µm | Mean c  contacts per 100 µm ±SD | Mean i  contacts per 100 µm ±SD | N  (animals) | Difference  Of Means | Adjusted p  Bonferroni | t |
| Bin 1: 0 - 50 | 1.2 ± 0.3 | 0.6 ± 0.2 | 4, 4 | 0.6 | 0.0811 | 2.886 |
| Bin 2: 50 -100 | 1.5 ± 0.5 | 0.9 ± 0.1 | 4, 4 | 0.6 | 0.0634 | 2.974 |
| Bin 3: 100 -150 | 1.6 ± 0.7 | 0.7 ± 0.3 | 4, 4 | 0.9 | 0.0018** | 4.120 |
| Control | | | | | | |
| Bin 1 vs Bin 2 |  |  |  | 0.3 | >0.9999 | 1.513 |
| Bin 1 vs Bin 3 |  |  |  | 0.4 | 0.6924 | 2.036 |
| Bin 2 vs Bin 3 |  |  |  | 0.1 | >0.9999 | 0.523 |
| Injured | | | | | | |
| Bin 1 vs Bin 2 |  |  |  | 0.3 | >0.9999 | 1.425 |
| Bin 1 vs Bin 3 |  |  |  | 0.2 | >0.9999 | 0.802 |
| Bin 2 vs Bin 3 |  |  |  | 0.1 | >0.9999 | 0.623 |
| **VGLUT1 surface density p25** | | | | | | |
| Bin 1: 0 - 50 | 1.2 ± 0.2 | 0.6 ± 0.1 | 4, 4 | 0.6 | 0.1439 | 2.676 |
| Bin 2: 50 -100 | 1.7 ± 0.4 | 1.0 ± 0.1 | 4, 4 | 0.7 | 0.0292* | 3.241 |
| Bin 3: 100 -150 | 1.7 ± 0.2 | 0.7 ± 0.2 | 4, 4 | 1.0 | 0.0002*** | 4.704 |
| Control | | | | | | |
| Bin 1 vs Bin 2 |  |  |  | 0.5 | 0.4501 | 2.223 |
| Bin 1 vs Bin 3 |  |  |  | 0.5 | 0.4620 | 2.212 |
| Bin 2 vs Bin 3 |  |  |  | 0.00 | >0.9999 | 0.011 |
| Injured | | | | | | |
| Bin 1 vs Bin 2 |  |  |  | 0.4 | >0.9999 | 1.658 |
| Bin 1 vs Bin 3 |  |  |  | 0.04 | >0.9999 | 0.183 |
| Bin 2 vs Bin 3 |  |  |  | 0.3 | >0.9999 | 1.474 |
| **VGLUT1 surface density p70** | | | | | | |
| Bin 1: 0 - 50 | 1.4 ± 0.2 | 0.8 ± 0.2 | 5, 5 | 0.6 | 0.0196* | 3.372 |
| Bin 2: 50 -100 | 1.8 ± 0.1 | 1.0 ± 0.3 | 5, 5 | 0.8 | 0.0010** | 4.279 |
| Bin 3: 100 -150 | 1.8 ± 0.4 | 0.9 ± 0.2 | 5, 5 | 0.9 | p<0.0001*** | 5.030 |
| Control | | | | | | |
| Bin 1 vs Bin 2 |  |  |  | 0.4 | 0.3515 | 2.326 |
| Bin 1 vs Bin 3 |  |  |  | 0.5 | 0.2832 | 2.413 |
| Bin 2 vs Bin 3 |  |  |  | 0.02 | >0.9999 | 0.876 |
| Injured | | | | | | |
| Bin 1 vs Bin 2 |  |  |  | 0.3 | >0.9999 | 1.419 |
| Bin 1 vs Bin 3 |  |  |  | 0.1 | >0.9999 | 0.755 |
| Bin 2 vs Bin 3 |  |  |  | 0.1 | >0.9999 | 0.664 |
