# Supplementary material for: Massive Loss of Proprioceptive Ia Synapses in Rat Spinal Motoneurons after Nerve Crush Injuries in the Postnatal Period
Source: eNeuro. 2023 Feb 14;10(2):ENEURO.0436-22.2023. doi: 10.1523/ENEURO.0436-22.2023 (PMC9948128; doi:10.1523/ENEURO.0436-22.2023)
Supplement: Figure 3-2 — Statistical table for changes in cell body VGluT1 contact number and density according to age and/or injury. Download Figure 3-2, DOCX file. [file enu-eN-NWR-0436-22-s06.docx]

**Extended data table Figure 3-2. Statistical table for changes in cell body VGLUT1 contact number and density according to age and/or injury.**

i = ipsilateral to injury; c = control contralateral to the injury (pooled data)

| **VGLUT1 number of contacts**  Normality, Shapiro-Wilk test: p = 0.404; passed normality test (α = 0.05)  Two-way ANOVA for injury and days postinjury (dpi)   - Postnatal date / days after injury (dpi): F_(2,20)_ = 9.669 p = 0.0012 - Injury: F_(1, 20)_ = 66.11 p < 0.0001 - Injury X dpi: F_(2,20)_ = 4.119 p = 0.0318   Multiple comparisons Bonferroni corrected t-tests | | | | | | |
| --- | --- | --- | --- | --- | --- | --- |
| Dates | Mean c  contacts ± SD | Mean i  contacts ± SD | N  (animals) | Difference  Of Means | Adjusted p  Bonferroni | t |
| 7 dpi (p17) | 16.4 ± 6.2 | 4.6 ± 2.0 | 4 | 11.7 | <0.001*** | 4.808 |
| 15 dpi (p25) | 14.6 ± 3.3 | 8.6 ± 3.1 | 4 | 6.0 | 0.022* | 2.476 |
| 60 dpi (p70) | 24.8 ± 1.0 | 9.4 ± 3.3 | 5 | 15.4 | <0.001*** | 7.067 |
| Control | | | | | | |
| p17 vs p25 |  |  |  | 1.7 | 1.000 | 0.709 |
| p17 vs p70 |  |  |  | 8.4 | 0.005** | 3.652 |
| p25 vs p70 |  |  |  | 10.2 | <0.001*** | 4.399 |
| Injured | | | | | | |
| p17 vs p25 |  |  |  | 4.0 | 0.361 | 1.623 |
| p17 vs p70 |  |  |  | 4.8 | 0.159 | 2.058 |
| p25 vs p70 |  |  |  | 0.8 | 1.000 | 0.347 |

| **VGLUT1 density**  Normality, Shapiro-Wilk test: p = 0.504; passed normality test (α = 0.05)  Two-way ANOVA for injury and days-post-injury (dpi)   - Postnatal date / days after injury (dpi): F_(2,20)_ = 0.235 p = 0.792 - Injury: F_(1, 20)_ = 61.245 p < 0.0001 - Injury X dpi: F_(2,20)_ = 1.9329 p = 0.171   Multiple comparisons Bonferroni corrected t-tests  (No pair-wise comparisons for because ANOVA showed no significance) | | | | | | |
| --- | --- | --- | --- | --- | --- | --- |
| Dates | Mean c  contacts per 100 µm^2^ | Mean i  contacts per 100 µm^2^ | N  (animals) | Difference  Of Means | Adjusted p  Bonferroni | t |
| 7 dpi (p17) | 0.57 ± 0.18 | 0.16 ± 0.07 | 4 | 0.4 | <0.001*** | 5.462 |
| 15 dpi (p25) | 0.49 ± 0.11 | 0.28 ± 0.09 | 4 | 0.2 | 0.032* | 2.814 |
| 60 dpi (p70) | 0.58 ± 0.09 | 0.21 ± 0.08 | 5 | 0.4 | <0.001*** | 5.389 |
