# Supplementary material for: Massive Loss of Proprioceptive Ia Synapses in Rat Spinal Motoneurons after Nerve Crush Injuries in the Postnatal Period
Source: eNeuro. 2023 Feb 14;10(2):ENEURO.0436-22.2023. doi: 10.1523/ENEURO.0436-22.2023 (PMC9948128; doi:10.1523/ENEURO.0436-22.2023)
Supplement: Figure 5-1 — Statistical table for changes in dendrite VGluT1 linear density according to age, injury, and distance from the cell body. Download Figure 5-1, DOCX file. [file enu-eN-NWR-0436-22-s07.docx]

**Extended data table Figure 5-1. Statistical table for changes in dendrites VGLUT1 linear density according to age, injury and distance from the cell body.**

i = ipsilateral to injury; c = control contralateral to the injury (individual motoneurons)

| Normality, Shapiro-Wilk test: p = 0.626; passed normality test (α = 0.05)  Two-way ANOVA for dpi and distance in injury and control.   - Dpi (control/injured): F_(5,337)_ = 131.5 p < 0.0001 - dendritic compartment: F_(2, 337)_ = 58.69 p < 0.0001 - interaction: F_(10, 337)_ = 1.674 p = 0.085 | | | | | | |
| --- | --- | --- | --- | --- | --- | --- |
| **VGLUT1 linear density p17** | | | | | | |
| Dendrite bins  µm | Mean c  contacts per 100 µm ±SD | Mean i  contacts per 100 µm ±SD | N  (MNs)  c , i | Difference  Of Means | Adjusted p  Bonferroni | t |
| Bin 1: 0 - 50 | 14.6 ± 4.3 | 6.0 ± 1.7 | 20, 20 | 8.6 | <0.0001*** | 9.002 |
| Bin 2: 50 -100 | 9.8 ± 3.2 | 4.4 ± 1.5 | 20, 20 | 5.4 | <0.0001*** | 5.638 |
| Bin 3: 100 -150 | 8.0 ± 4.1 | 3.3 ± 2.3 | 20, 19 | 4.7 | <0.0002*** | 4.384 |
| Control | | | | | | |
| Bin 1 vs Bin 2 |  |  |  | 4.9 | <0.0001*** | 5.071 |
| Bin 1 vs Bin 3 |  |  |  | 6.7 | <0.0001*** | 6.654 |
| Bin 2 vs Bin 3 |  |  |  | 1.8 | >0.9999 | 1.793 |
| Injured | | | | | | |
| Bin 1 vs Bin 2 |  |  |  | 2.7 | >0.9999 | 1.707 |
| Bin 1 vs Bin 3 |  |  |  | 1.6 | 0.1307 | 2.639 |
| Bin 2 vs Bin 3 |  |  |  | 1.1 | >0.9999 | 1.058 |
| **VGLUT1 linear density p25** | | | | | | |
| Bin 1: 0 - 50 | 14.5 ± 4.3 | 7.3 ± 2.2 | 19,19 | 7.2 | <0.0001*** | 7.310 |
| Bin 2: 50 -100 | 9.9 ± 3.3 | 5.2 ± 2.3 | 19,19 | 4.7 | <0.0001*** | 4.794 |
| Bin 3: 100 -150 | 8.5 ± 2.8 | 2.8 ± 1.6 | 19,19 | 5.7 | <0.0001*** | 7.950 |
| Control | | | | | | |
| Bin 1 vs Bin 2 |  |  |  | 4.6 | <0.0001*** | 4.631 |
| Bin 1 vs Bin 3 |  |  |  | 5.9 | <0.0001*** | 5.305 |
| Bin 2 vs Bin 3 |  |  |  | 1.4 | >0.9999 | 1.230 |
| Injured | | | | | | |
| Bin 1 vs Bin 2 |  |  |  | 4.5 | 0.5259 | 2.116 |
| Bin 1 vs Bin 3 |  |  |  | 2.1 | 0.0003*** | 4.347 |
| Bin 2 vs Bin 3 |  |  |  | 2.4 | 0.3109 | 2.324 |
| **VGLUT1 linear density p70**  Normality, Shapiro-Wilk test: p = 0.831; passed normality test (α = 0.05)  One-Way ANOVA: F_(5, 138)_ = 69.284 p < 0.001 | | | | | | |
| Bin 1: 0 - 50 | 20.4 ± 3.8 | 10.7 ± 2.6 | 26, 25 | 9.8 | <0.0001*** | 11.48 |
| Bin 2: 50 -100 | 14.0 ± 3.3 | 7.2 ± 2.0 | 26, 25 | 6.8 | <0.0001*** | 7.944 |
| Bin 3: 100 -150 | 11.0 ± 3.6 | 5.5 ± 3.5 | 26, 25 | 3.8 | <0.0001*** | 5.517 |
| Control | | | | | | |
| Bin1 vs Bin2 |  |  |  | 6.5 | <0.0001*** | 7.619 |
| Bin 1 vs Bin 3 |  |  |  | 9.5 | <0.0001*** | 10.03 |
| Bin 2 vs Bin 3 |  |  |  | 3.0 | 0.0253* | 3.166 |
| Injured | | | | | | |
| Bin 1 vs Bin 2 |  |  |  | 3.5 | 0.0007*** | 4.120 |
| Bin 1 vs Bin 3 |  |  |  | 5.2 | <0.0001*** | 5.789 |
| Bin 2 vs Bin 3 |  |  |  | 1.7 | 0.9733 | 1.852 |
