# Supplementary material for: Massive Loss of Proprioceptive Ia Synapses in Rat Spinal Motoneurons after Nerve Crush Injuries in the Postnatal Period
Source: eNeuro. 2023 Feb 14;10(2):ENEURO.0436-22.2023. doi: 10.1523/ENEURO.0436-22.2023 (PMC9948128; doi:10.1523/ENEURO.0436-22.2023)
Supplement: Figure 5-2 — Statistical table for changes in dendrites VGluT1 linear density according to age, injury, and distance from the cell body. Download Figure 5-2, DOCX file. [file enu-eN-NWR-0436-22-s08.docx]

**Extended data table Figure 5-2. Statistical table for changes in dendrites VGLUT1 linear density according to age, injury and distance from the cell body.**

i = ipsilateral to injury; c = control contralateral to injury (pooled data per animal average)

| Normality, Shapiro-Wilk test: p > 0.1 in all data sets; pass normality test (α = 0.05)  Two-way ANOVA for dpi and distance in injury and control.   - dpi (control/injured): F_(2,60)_ = 28.77 p < 0.0001 - dendritic compartment F_(5, 60)_ = 67.38 p < 0.0001 - interaction: F_(10, 60)_ = 1.021 p = 0.4372   Multiple comparisons Bonferroni corrected t-tests | | | | | | |
| --- | --- | --- | --- | --- | --- | --- |
| **VGLUT1 linear density p17** | | | | | | |
| Dendrite bins  µm | Mean c  contacts per 100 µm | Mean i  contacts per 100 µm | N  (animals) | Difference  Of Means | Adjusted p  Bonferroni | t |
| Bin 1: 0 - 50 | 14.9 ± 3.1 | 6.0 ± 1.4 | 4, 4 | 8.9 | <0.0001*** | 6.365 |
| Bin 2: 50 -100 | 9.8 ± 1.7 | 4.4 ± 0.6 | 4, 4 | 6.6 | 0.0003*** | 3.901 |
| Bin 3: 100 -150 | 8.4 ± 3.5 | 2.8 ± 1.0 | 4, 4 | 5.6 | 0.0031** | 3.956 |
| Control | | | | | | |
| Bin 1 vs Bin 2 |  |  |  | 5.1 | 0.0088** | 3.629 |
| Bin 1 vs Bin 3 |  |  |  | 12.12 | <0.0001*** | 4.672 |
| Bin 2 vs Bin 3 |  |  |  | 1.5 | >0.9999 | 1.043 |
| Injured | | | | | | |
| Bin 1 vs Bin 2 |  |  |  | 1.6 | >0.9999 | 1.165 |
| Bin 1 vs Bin 2 |  |  |  | 3.2 | 0.4098 | 2.262 |
| Bin 2 vs Bin 3 |  |  |  | 1.5 | >0.9999 | 1.097 |
| **VGLUT1 linear density p25** | | | | | | |
| Bin 1: 0 - 50 | 14.2 ± 3.3 | 7.4 ± 0.9 | 4, 4 | 6.9 | <0.0001*** | 4.883 |
| Bin 2: 50 -100 | 10.1 ± 1.7 | 5.3 ± 0.8 | 4, 4 | 4.8 | 0.0170* | 3.419 |
| Bin 3: 100 -150 | 9.6 ± 2.7 | 2.6 ± 0.7 | 4, 4 | 7.0 | <0.0001*** | 5.011 |
| Control | | | | | | |
| Bin1 vs Bin2 |  |  |  | 4.2 | 0.0674 | 2.952 |
| Bin 1 vs Bin 3 |  |  |  | 4.6 | 0.0277* | 3.259 |
| Bin 2 vs Bin 3 |  |  |  | 0.4 | >0.9999 | 0.3065 |
| Injured | | | | | | |
| Bin 1 vs Bin 2 |  |  |  | 2.1 | >0.9999 | 1.488 |
| Bin 1 vs Bin 2 |  |  |  | 4.8 | 0.0188* | 3.386 |
| Bin 2 vs Bin 3 |  |  |  | 2.7 | 0.9377 | 1.898 |
| **VGLUT1 linear density p70** | | | | | | |
| Bin 1: 0 - 50 | 20.5 ± 1.6 | 10.7 ± 1.6 | 5, 5 | 9.8 | <0.0001*** | 7.759 |
| Bin 2: 50 -100 | 14.0 ± 0.4 | 7.2 ± 1.7 | 5, 5 | 6.8 | <0.0001*** | 5.367 |
| Bin 3: 100 -150 | 10.8 ± 2.5 | 5.5 ± 2.6 | 5, 5 | 5.3 | 0.0013** | 4.204 |
| Control | | | | | | |
| Bin 1 vs Bin 2 |  |  |  | 6.5 | <0.0001*** | 5.163 |
| Bin 1 vs Bin 3 |  |  |  | 9.7 | <0.0001*** | 7.685 |
| Bin 2 vs Bin 3 |  |  |  | 3.2 | 0.2153 | 2.522 |
| Injured | | | | | | |
| Bin 1 vs Bin 2 |  |  |  | 3.5 | 0.1113 | 2.771 |
| Bin 1 vs Bin 2 |  |  |  | 5.2 | 0.0017** | 4.130 |
| Bin 2 vs Bin 3 |  |  |  | 1.7 | >0.9999 | 1.358 |
