# Supplementary material for: Massive Loss of Proprioceptive Ia Synapses in Rat Spinal Motoneurons after Nerve Crush Injuries in the Postnatal Period
Source: eNeuro. 2023 Feb 14;10(2):ENEURO.0436-22.2023. doi: 10.1523/ENEURO.0436-22.2023 (PMC9948128; doi:10.1523/ENEURO.0436-22.2023)
Supplement: Figure 5-3 — Statistical table for changes in dendrite VGluT1 surface density according to age, injury, and distance from the cell body. Download Figure 5-3, DOCX file. [file enu-eN-NWR-0436-22-s15.docx]

**Extended data table Figure 5-3. Statistical table for changes in dendrite VGLUT1 surface density according to age, injury and distance from the cell body.**

i = ipsilateral to injury; c = control contralateral to injury (individual motoneurons)

| Normality, Shapiro-Wilk test: p > 0.8 in all data sets; pass normality test (α = 0.05)  Two-way ANOVA for dpi and distance in injury and control.   - dpi (control/injured): F_(2, 337)_ = 10.550 p < 0.001 - dendritic compartment F_(5,337)_ = 44.11 p = 0.0067 - interaction: F_(10, 337)_ = 0.2763 p = 0.986   Multiple comparisons Bonferroni corrected t-tests | | | | | | |
| --- | --- | --- | --- | --- | --- | --- |
| **VGLUT1 surface density p17** | | | | | | |
| Dendrite bins  µm | Mean c  contacts per 100 µm ±SD | Mean i  contacts per 100 µm ±SD | N  (MNs) | Difference  Of Means | Adjusted p  Bonferroni | t |
| Bin 1: 0 - 50 | 1.2 ± 0.4 | 0.6 ± 0.2 | 20, 20 | 0.6 | 0.0036** | 3.717 |
| Bin 2: 50 -100 | 1.5 ± 0.6 | 0.9 ± 0.3 | 20, 20 | 0.6 | 0.0015** | 3.937 |
| Bin 3: 100 -150 | 1.6 ± 0.9 | 0.8 ± 0.5 | 19, 19 | 0.8 | 0.0003*** | 4.331 |
| Control | | | | | | |
| Bin 1 vs Bin 2 |  |  |  | 0.3 | 0.5169 | 2.124 |
| Bin 1 vs Bin 3 |  |  |  | 0.4 | 0.2684 | 2.380 |
| Bin 2 vs Bin 3 |  |  |  | 0.1 | >0.9999 | 0.378 |
| Injured | | | | | | |
| Bin 1 vs Bin 2 |  |  |  | 0.3 | 0.8682 | 1.903 |
| Bin 1 vs Bin 3 |  |  |  | 0.2 | >0.9999 | 0.999 |
| Bin 2 vs Bin 3 |  |  |  | 0.1 | >0.9999 | 0.690 |
| **VGLUT1 surface density p25** | | | | | | |
| Bin 1: 0 - 50 | 1.2 ± 0.4 | 0.6 ± 0.2 | 19, 19 | 0.6 | 0.0073** | 3.522 |
| Bin 2: 50 -100 | 1.6 ± 0.9 | 1.0 ± 0.5 | 19, 19 | 0.7 | 0.0004*** | 4.268 |
| Bin 3: 100 -150 | 1.7 ± 0.5 | 0.8 ± 0.5 | 19, 16 | 1.0 | <0.0001*** | 5.068 |
| Control | | | | | | |
| Bin 1 vs Bin 2 |  |  |  | 0.5 | 0.0604 | 2.897 |
| Bin 1 vs Bin 3 |  |  |  | 0.5 | 0.0624 | 2.886 |
| Bin 2 vs Bin 3 |  |  |  | 0.1 | >0.9999 | 0.338 |
| Injured | | | | | | |
| Bin 1 vs Bin 2 |  |  |  | 0.4 | 0.4834 | 2.151 |
| Bin 1 vs Bin 3 |  |  |  | 0.1 | >0.9999 | 0.497 |
| Bin 2 vs Bin 3 |  |  |  | 0.3 | >0.9999 | 1.444 |
| **VGLUT1 surface density p70** | | | | | | |
| Bin 1: 0 - 50 | 1.4 ± 0.3 | 0.7 ± 0.2 | 26, 25 | 0.7 | <0.0001*** | 4.814 |
| Bin 2: 50 -100 | 1.8 ± 0.5 | 1.0 ± 0.4 | 26, 25 | 0.9 | <0.0001*** | 6.068 |
| Bin 3: 100 -150 | 1.8 ± 0.8 | 0.9 ± 0.5 | 26, 25 | 0.9 | <0.0001*** | 5.159 |
| Control | | | | | | |
| Bin 1 vs Bin 2 |  |  |  | 0.4 | 0.0321* | 3.095 |
| Bin 1 vs Bin 3 |  |  |  | 0.5 | 0.0748 | 2.827 |
| Bin 2 vs Bin 3 |  |  |  | 0.02 | >0.9999 | 0.098 |
| Injured | | | | | | |
| Bin 1 vs Bin 2 |  |  |  | 0.3 | >0.9999 | 1.765 |
| Bin 1 vs Bin 3 |  |  |  | 0.2 | >0.9999 | 1.534 |
| Bin 2 vs Bin 3 |  |  |  | 0.01 | >0.9999 | 0.081 |
