# Supplementary material for: Massive Loss of Proprioceptive Ia Synapses in Rat Spinal Motoneurons after Nerve Crush Injuries in the Postnatal Period
Source: eNeuro. 2023 Feb 14;10(2):ENEURO.0436-22.2023. doi: 10.1523/ENEURO.0436-22.2023 (PMC9948128; doi:10.1523/ENEURO.0436-22.2023)
Supplement: Figure 5-5 — Statistical table for changes in the number of VGluT1 contacts with age in each dendrite compartment in controls. Download Figure 5-5, DOCX file. [file enu-eN-NWR-0436-22-s09.docx]

**Extended data table Figure 5-5. Statistical table for changes in number of VGLUT1 contacts with age in each dendrite compartment in controls**

| Normality, Shapiro-Wilk test: p > 0.5 in all data sets; pass normality test (α = 0.05)  Two-way ANOVA age and distance to cell body in control.   - age (control): F_(2, 154)_ = 22.33 p < 0.0001 - dendritic compartment F_(2, 154)_ = 62.84 p < 0.0001 - interaction: F_(4, 154)_ = 0.5364 p = 0.7092   Multiple comparisons Bonferroni corrected t-tests | | | | |
| --- | --- | --- | --- | --- |
| Age (postnatal days) | Predicted mean  difference | SE  of difference | Adjusted p  Bonferroni | t |
| Distance 0 to 50 µm from cell body. Control | | | | |
| p17 vs p25 | 0.26 | 1.29 | >0.9999 | 0.2014 |
| p17 vs p70 | 4.97 | 1.18 | 0.0001*** | 4.203 |
| p25 vs p70 | 4.71 | 1.21 | 0.0004*** | 3.907 |
| Distance 50 to 100 µm from cell body. Control | | | | |
| p17 vs p25 | 0.14 | 1.15 | >0.9999 | 0.1255 |
| p17 vs p70 | 4.19 | 1.08 | 0.0005*** | 3.882 |
| p25 vs p70 | 4.05 | 1.10 | 0.0009*** | 3.625 |
| Distance 100 to 150 µm from cell body. Control | | | | |
| p17 vs p25 | 0.56 | 1.36 | >0.9999 | 0.4147 |
| p17 vs p70 | 2.97 | 1.24 | 0.0522 | 2.404 |
| p25 vs p70 | 2.41 | 1.36 | 0.2349 | 1.772 |
