# Supplementary material for: Massive Loss of Proprioceptive Ia Synapses in Rat Spinal Motoneurons after Nerve Crush Injuries in the Postnatal Period
Source: eNeuro. 2023 Feb 14;10(2):ENEURO.0436-22.2023. doi: 10.1523/ENEURO.0436-22.2023 (PMC9948128; doi:10.1523/ENEURO.0436-22.2023)
Supplement: Figure 5-6 — Statistical table for changes in number of VGluT1 contacts with age in each dendrite compartment after injury. Download Figure 5-6, DOCX file. [file enu-eN-NWR-0436-22-s10.docx]

**Extended data table Figure 5-6. Statistical table for changes in number of VGLUT1 contacts with age in each dendrite compartment in injury**

| Normality, Shapiro-Wilk test: p > 0.5 in all data sets; pass normality test (α = 0.05)  Two-way ANOVA age and distance to cell body in injury.   - age (control): F_(2, 171)_ = 36.12 p < 0.0001 - dendritic compartment F_(2, 171)_ = 47.04 p < 0.0001 - interaction: F_(4, 171)_ = 1.908 p = 01112   Multiple comparisons Bonferroni corrected t-tests | | | | |
| --- | --- | --- | --- | --- |
| Age (postnatal days) | Predicted mean  difference | SE  of difference | Adjusted p  Bonferroni | t |
| Distance 0 to 50 µm from cell body. Injured | | | | |
| p17 vs p25 | 1.28 | 0.74 | >0.9999 | 1.741 |
| p17 vs p70 | 4.68 | 0.69 | <0.0001*** | 6.805 |
| p25 vs p70 | 3.40 | 0.70 | <0.0001*** | 4.875 |
| Distance 50 to 100 µm from cell body. Injured | | | | |
| p17 vs p25 | 0.83 | 0.73 | 0.7734 | 1.135 |
| p17 vs p70 | 2.79 | 0.69 | 0.0002*** | 4.048 |
| p25 vs p70 | 2.0 | 0.70 | 0.0174* | 2.795 |
| Distance 100 to 150 µm from cell body. Injured | | | | |
| p17 vs p25 | 0.46 | 0.82 | >0.9999 | 0.559 |
| p17 vs p70 | 2.22 | 0.78 | 0.0142* | 2.861 |
| p25 vs p70 | 2.68 | 0.76 | 0.0017** | 3.521 |
