# Supplementary material for: Massive Loss of Proprioceptive Ia Synapses in Rat Spinal Motoneurons after Nerve Crush Injuries in the Postnatal Period
Source: eNeuro. 2023 Feb 14;10(2):ENEURO.0436-22.2023. doi: 10.1523/ENEURO.0436-22.2023 (PMC9948128; doi:10.1523/ENEURO.0436-22.2023)
Supplement: Figure 6-1 — Statistical table for changes in number of PV cells in the L4 DRG at 60 dpi. Download Figure 6-1, DOCX file. [file enu-eN-NWR-0436-22-s11.docx]

**Extended data table Figure 6-1. Statistical table for changes in number of PV cells in the L4 DRG at 60 days postinjury**

| **Number of PV cells**  Normality, Shapiro-Wilk test:   - Control distribution p = 0.2408 - Injured distribution p = 0.0770   T-test, two-tailed, equal variances  t = 1.175; df = 12, p = 0.2628 | | | |
| --- | --- | --- | --- |
| Control  ±S.D. | Injured  ±S.D. | Difference between means ± S.E.M | 95% CI |
| 85.6 ±22.4 | 68.2 ±30.7 | -17.5 ±14.9 | -49.8 to 14.9 |
| **Percentage of PV cells to all NeuN cells**  Normality, Shapiro-Wilk test:   - Control distribution p = 0.8766 - Injured distribution p = 0.1237   T-test, two-tailed, unequal variances  t = 1.1.628; df = 12, p = 0.1295 | | | |
| 25.7 ±2.4 | 20.3 ±7.7 | -5.4 ±3.3 | -12.5 to 1.8 |
