# Supplementary material for: Massive Loss of Proprioceptive Ia Synapses in Rat Spinal Motoneurons after Nerve Crush Injuries in the Postnatal Period
Source: eNeuro. 2023 Feb 14;10(2):ENEURO.0436-22.2023. doi: 10.1523/ENEURO.0436-22.2023 (PMC9948128; doi:10.1523/ENEURO.0436-22.2023)
Supplement: Figure 7-1 — Statistical table for changes in microglia number after nerve crush injury at P10. Download Figure 7-1, DOCX file. [file enu-eN-NWR-0436-22-s12.docx]

**Extended data table Figure 7-1. Statistical table for changes in microglia number after nerve crush injury at p10.**

| **VGLUT1 contacts**  Normality, Shapiro-Wilk test: Control p = 0.9571; Injured p = 0.9454  passed normality test (α = 0.05)  Two-way ANOVA for injury and days postinjury (dpi)   - Postnatal date / days after injury (dpi): F_(5,20)_ = 51.63 p < 0.0001 - Injury: F_(5, 20)_ = 211.9 p < 0.0001 - Injury X dpi: F_(1,20)_ = 738.7 p < 0.0001   Multiple comparisons Bonferroni corrected t-tests | | | | | | | |
| --- | --- | --- | --- | --- | --- | --- | --- |
| Dates | Microglia # per 10^6^ µm^3^ | | N  (animals) | Difference  Of Means | Ratio  ± SEM | Adjusted p  Bonferroni | t |
|  | Control side ± SD | Experimental  ± SD |  |  |  |  |  |
| p13  (3 dpi) | 9.3 ± 0.2 | 18.6 ± 0.4 | 2 | 9.3 | 2.0 ± 0.06 | <0.0001*** | 14.36 |
| p15  (5 dpi) | 10.0 ± 0.4 | 21.0 ± 0.7 | 3 | 11.0 | 2.1 ± 0.06 | <0.0001*** | 20.71 |
| p17  (7 dpi) | 7.2 ± 0.4 | 16.6 ± 2.2 | 3 | 9.4 | 2.3 ± 0.11 | <0.0001*** | 17.68 |
| p24  (14 dpi) | 6.2 ± 0.4 | 11.2 ± 0.0 | 3 | 5.0 | 1.8 ± 0.10 | <0.0001*** | 9.370 |
| p31  (21 dpi) | 5.7 ± 0.1 | 8.1± 0.1 | 3 | 2.4 | 1.5 ± 0.01 | 0.0015** | 4.451 |
| p70  (60 dpi) | 4.0 ± 0.4 | 5.1 ± 0.4 | 2 | 1.1 | 1.3 ± 0.02 | 0.6019 | 1.723 |
| Control side changes with age | | | | | | | |
| p13 vs p15 |  |  | 2 , 3 | +0.68 |  | >0.9999 | 1.150 |
| p13 vs p17 |  |  | 2 , 3 | -2.14 |  | 0.0267* | 3.602 |
| p13 vs p24 |  |  | 2 , 3 | -3.11 |  | 0.0006*** | 5.237 |
| p13 vs p31 |  |  | 2 , 3 | -3.61 |  | <0.0001*** | 6.073 |
| p13 vs p70 |  |  | 2 , 2 | -5.32 |  | <0.0001*** | 8.181 |
| p15 vs p17 |  |  | 3 , 3 | -2.82 |  | 0.0005*** | 5.312 |
| p15 vs p24 |  |  | 3 , 3 | -3.79 |  | <0.0001*** | 7.141 |
| p15 vs p31 |  |  | 3 , 3 | -4.30 |  | <0.0001*** | 8.075 |
| p15 vs p70 |  |  | 3 , 2 | -6.01 |  | <0.0001*** | 10.11 |
| p17 vs p24 |  |  | 3 , 3 | -0.97 |  | >0.9999 | 1.828 |
| p17 vs p31 |  |  | 3 , 3 | -1.47 |  | 0.1799 | 2.763 |
| p17 vs p70 |  |  | 3 , 2 | -3.18 |  | 0.0005*** | 5.359 |
| p24 vs p31 |  |  | 3 , 3 | -0.50 |  | >0.9999 | 0.9347 |
| p24 vs p70 |  |  | 3 , 2 | -2.21 |  | 0.0201* | 3.724 |
| p31 vs p70 |  |  | 3 , 2 | -1.72 |  | 0.1364 | 2.888 |
| Injured side changes with age and time after nerve crush | | | | | | | |
| p13 vs p15 |  |  | 2 , 3 | +2.34 |  | 0.0122** | 3.940 |
| p13 vs p17 |  |  | 2 , 3 | -2.09 |  | 0.0326* | 3.516 |
| p13 vs p24 |  |  | 2 , 3 | -7.48 |  | <0.0001*** | 12.59 |
| p13 vs p31 |  |  | 2 , 3 | -10.59 |  | <0.0001*** | 17.82 |
| p13 vs p70 |  |  | 2 , 2 | -13.55 |  | <0.0001*** | 20.82 |
| p15 vs p17 |  |  | 3 , 3 | -4.43 |  | <0.0001*** | 8.335 |
| p15 vs p24 |  |  | 3 , 3 | -9.82 |  | <0.0001*** | 18.48 |
| p15 vs p31 |  |  | 3 , 3 | -12.93 |  | <0.0001*** | 24.33 |
| p15 vs p70 |  |  | 3 , 2 | -15.89 |  | <0.0001*** | 26.74 |
| p17 vs p24 |  |  | 3 , 3 | -5.39 |  | <0.0001*** | 10.14 |
| p17 vs p31 |  |  | 3 , 3 | -8.50 |  | <0.0001*** | 16.00 |
| p17 vs p70 |  |  | 3 , 2 | -11.46 |  | <0.0001*** | 19.29 |
| p24 vs p31 |  |  | 3 , 3 | -3.11 |  | 0.0001*** | 5.854 |
| p24 vs p70 |  |  | 3 , 2 | -6.07 |  | <0.0001*** | 10.22 |
| p31 vs p70 |  |  | 3 , 2 | -3.00 |  | 0.0011** | 4.982 |
