# Supplementary material for: Massive Loss of Proprioceptive Ia Synapses in Rat Spinal Motoneurons after Nerve Crush Injuries in the Postnatal Period
Source: eNeuro. 2023 Feb 14;10(2):ENEURO.0436-22.2023. doi: 10.1523/ENEURO.0436-22.2023 (PMC9948128; doi:10.1523/ENEURO.0436-22.2023)
Supplement: Figure 7-2 — Statistical table for changes in ventral horn size with postnatal developmental maturation. Download Figure 7-2, DOCX file. [file enu-eN-NWR-0436-22-s13.docx]

**Extended data table Figure 7-2. Statistical table for changes in ventral horn size with postnatal developmental maturation**

| Normality, Shapiro-Wilk test: p > 0.9 in all data sets; pass normality test (α = 0.05)  One-way ANOVA for age F_(5, 88)_ = 76.14; p < 0.0001  Multiple comparisons Bonferroni corrected t-tests | | | | | |
| --- | --- | --- | --- | --- | --- |
| Age  (postnatal days) | N1, N2  (only sections from L4 or L5) | Mean  Difference (µm^3^) | Percentage increase  (%) | Adjusted p  Bonferroni | t |
| p13 vs p15 | 12, 18 | 68,713 | 16.4 | 0.0414* | 3.080 |
| p13 vs p17 | 12, 18 | 136,985 | 32.7 | <0.0001*** | 6.140 |
| p13 vs p24 | 12, 18 | 253,645 | 60.5 | <0.0001*** | 11.37 |
| p13 vs p31 | 12, 18 | 313,240 | 74.7 | <0.0001*** | 14.04 |
| p13 vs p70 | 12, 10 | 354,672 | 84.6 | <0.0001*** | 13.84 |
| p15 vs p17 | 18, 18 | 68,272 | 14.0 | 0.0142* | 3.421 |
| p15 vs p24 | 18, 18 | 184,933 | 37.9 | <0.0001*** | 9.268 |
| p15 vs p31 | 18, 18 | 244,527 | 50.1 | <0.0001*** | 12.25 |
| p15 vs p70 | 18, 10 | 285,960 | 58.6 | <0.0001*** | 12.11 |
| p17 vs p24 | 18, 18 | 116,660 | 21.0 | <0.0001*** | 5.846 |
| p17 vs p31 | 18, 18 | 176,255 | 31.7 | <0.0001*** | 8.833 |
| p17 vs p70 | 18, 10 | 217,688 | 39.1 | <0.0001*** | 9.220 |
| p24 vs p31 | 18, 18 | 59,595 | 8.9 | 0.0548 | 2.987 |
| p24 vs p70 | 18, 10 | 101,027 | 15.0 | 0.0007*** | 4.279 |
| p31 vs p70 | 18, 10 | 41,433 | 5.7 | >0.9999 | 1.755 |
